# A strategy of local hydrogen capture and catalytic hydrogenation for enhanced therapy of chronic liver diseases

**DOI:** 10.7150/thno.80494

**Published:** 2023-04-23

**Authors:** Geru Tao, Feng Liu, Zhaokui Jin, Boyan Liu, Hao Wang, Daosheng Li, Wei Tang, Yuan Chen, Qianjun He, Shucun Qin

**Affiliations:** 1 Key Laboratory of Major Diseases and Hydrogen Medical Translational Applications in Universities of Shandong Province & Key Laboratory of Hydrogen Biomedical Research of Health Commission of Shandong Province, The Second Affiliated Hospital of Shandong First Medical University & Shandong Academy of Medical Sciences, Tai'an 271000, China.; 2 Taishan Institute for Hydrogen Biomedical Research, Shandong First Medical University & Shandong Academy of Medical Sciences, Tai'an 271000, China; 3 School of Biomedical Engineering, Guangzhou Medical University, Guangdong, 511495 China.; 4 Pathology Department of Tai'an City Central Hospital, Tai'an 271016, China.; 5 Key Laboratory of Human-Machine-Intelligence Synergic System, Research Center for Neural Engineering, Shenzhen Institutes of Advanced Technology, Chinese Academy of Sciences, Shenzhen 518055, Guangdong, China.; 6 Shanghai Key Laboratory of Hydrogen Science & Center of Hydrogen Science, School of Materials Science and Engineering, Shanghai Jiao Tong University, Shanghai 200240, China.; 7 Shenzhen Research Institute, Shanghai Jiao Tong University, Shenzhen 518057, China.

**Keywords:** catalysis, controlled release, targeted delivery, nanomedicine, hydrogen therapy, chronic liver diseases

## Abstract

**Background:** Chronic liver diseases (CLD) frequently derive from hepatic steatosis, inflammation and fibrosis, and become a leading inducement of cirrhosis and hepatocarcinoma. Molecular hydrogen (H_2_) is an emerging wide-spectrum anti-inflammatory molecule which is able to improve hepatic inflammation and metabolic dysfunction, and holds obvious advantages in biosafety over traditional anti-CLD drugs, but existing H_2_ administration routes cannot realize the liver-targeted high-dose delivery of H_2_, severely limiting its anti-CLD efficacy.

**Method:** In this work, a concept of local hydrogen capture and catalytic hydroxyl radical (·OH) hydrogenation is proposed for CLD treatment. The mild and moderate non-alcoholic steatohepatitis (NASH) model mice were intravenously injected with PdH nanoparticles firstly, and then daily inhaled 4% hydrogen gas for 3 h throughout the whole treatment period. After the end of treatment, glutathione (GSH) was intramuscularly injected every day to assist the Pd excretion.

**Results:** In vitro and in vivo proof-of-concept experiments have confirmed that Pd nanoparticles can accumulate in liver in a targeted manner post intravenous injection, and play a dual role of hydrogen captor and ·OH filter to locally capture/store the liver-passing H_2_ during daily hydrogen gas inhalation and rapidly catalyze the ·OH hydrogenation into H_2_O. The proposed therapy significantly improves the outcomes of hydrogen therapy in the prevention and treatment of NASH by exhibiting a wide range of bioactivity including the regulation of lipid metabolism and anti-inflammation. Pd can be mostly eliminated after the end of treatment under the assistance of GSH.

**Conclusion:** Our study verified a catalytic strategy of combining PdH nanoparticles and hydrogen inhalation, which exhibited enhanced anti-inflammatory effect for CLD treatment. The proposed catalytic strategy will open a new window to realize safe and efficient CLD treatment.

## Introduction

Chronic liver diseases (CLD) are characterized by progressive deterioration of liver functions which often results from hepatic steatosis, inflammation and fibrosis and eventually leads to cirrhosis and even hepatic carcinoma 1,2. CLD mainly includes viral hepatitis, alcoholic liver disease (ALD), non-alcoholic fatty liver disease (NAFLD) and non-alcoholic steatohepatitis (NASH). In spite of various types of CLD, chronic liver inflammation is a crucial factor in the pathogenesis and progression of CLD, and therefore in-time and efficient anti-inflammation is vitally important to CLD prevention and treatment, but still challenging 3-6. NASH is an underrecognized disease in clinical practice with increasing prevalence. It is strongly associated with metabolic syndrome, type 2 diabetes, and cardiovascular diseases. About 20% of NASH patients will develop cirrhosis, and NASH is predicted to become the leading indication for liver transplants in the US 7. Traditional anti-inflammatory drugs frequently bring toxic side effects and increase the metabolic burden of liver 8,9. The development of broad-spectrum anti-inflammation strategies and safe anti-inflammatory drugs is urgently needed for prevention and treatment of CLD.

Molecular hydrogen (H_2_) is a highly-biosafe broad-spectrum anti-inflammatory agent that is able to neutralize cytotoxic oxygen radicals including ·OH under the catalytic hydrogenation of Fe-porphyrin 10-12, exhibiting obvious therapeutic and non-toxic effects in many inflammation-related diseases such as hepatitis 13-23. Typically, several clinical trials consistently indicated that both inflammation and metabolic dysfunction in CLD can be improved by drinking hydrogen-rich water (HRW) 24,25. But the daily drinking amount of water is limited and the saturated solubility of H_2_ is considerably low (1.6 mg/L at 1 atm), leading to a relatively low dose of H_2_ and consequently limited therapeutic outcomes 26,27. Hydrogen gas inhalation is another popular hydrogen administration route for anti-CLD 28, but its high dispersion and low solubility cause inefficient accumulation in liver 29, severely limiting the anti-CLD efficacy of hydrogen gas inhalation therapy. The liver-targeted high-dose delivery of H_2_ remains a challenge for H_2_ therapy of CLD.

**Figure 1:**
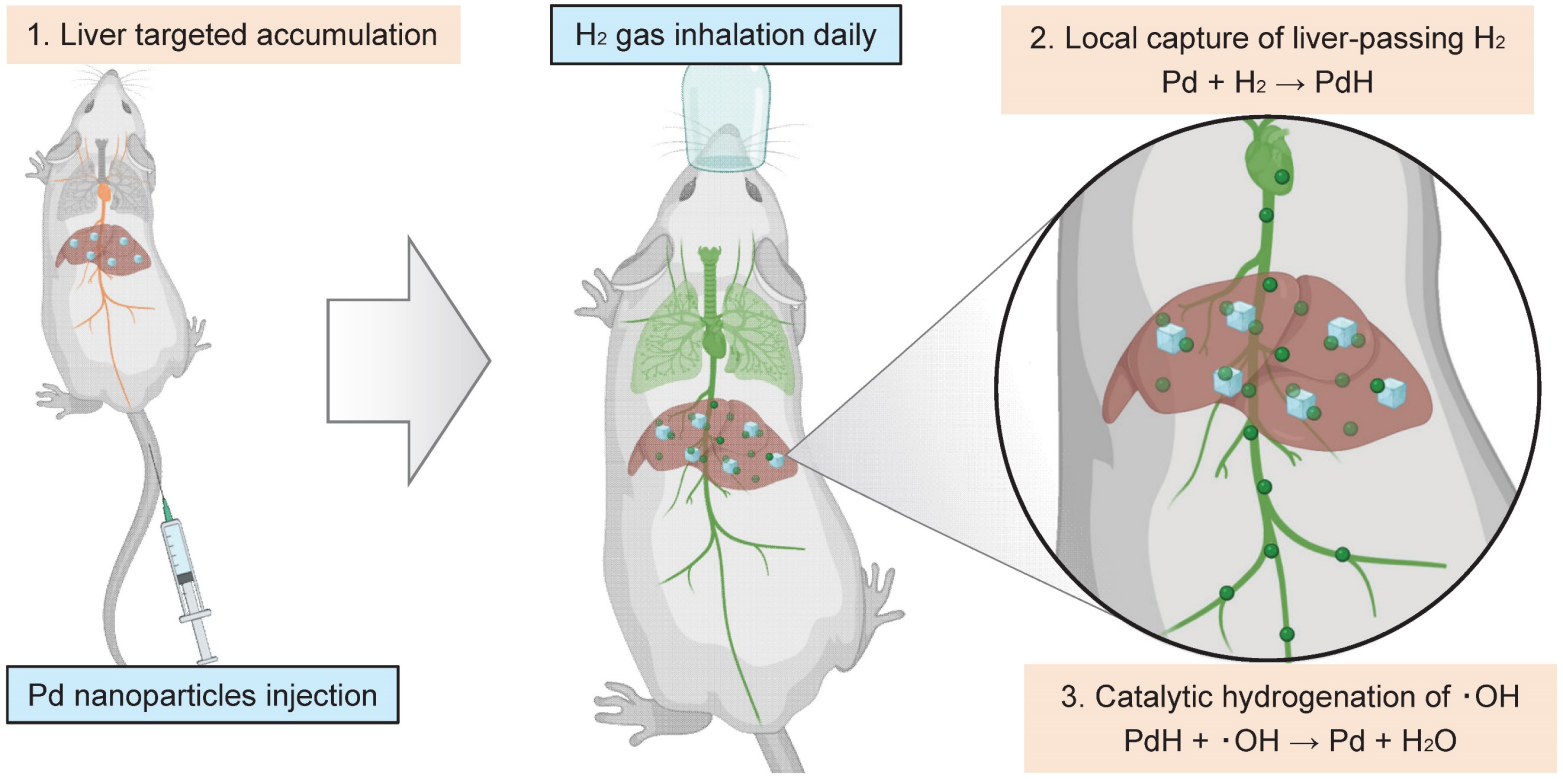
Schematic illustration of the therapeutic strategy and mechanism for local hydrogen capture and catalytic ‧OH hydrogenation with Pd nanoparticles in liver.

Nanomaterials have proven to provide enhanced therapeutic effect in wound healing and cancer chemotherapy 30,31. In recent years, nanomedicines present excellent biosafety, enhanced specificity in oxidative stress induced diseases which provide promising therapeutic strategies 32-35. Significantly, palladium nanocrystals possess excellent hydrogen storage capacity and catalytic properties for hydrogenation reaction at relatively high temperature and normal pressure 22,36-39. Typically, Pd is commonly used as a high-temperature hydrogenation catalyst in industry based on the strong coordination between Pd and H_2_. Therefore, we here speculated that the hydrogenation of Pd nanocrystals possibly occurs *in vivo* (at 37°C and at normal pressure). Moreover, by modulating the size and surface properties of Pd nanoparticles, their accumulation in the liver can be specifically targeted and greatly enhanced, potentially promoting hydrogen therapy outcomes. In this work, we proposed a therapeutic concept of local hydrogen capture and catalytic hydrogenation for liver-targeted high-dose delivery of H_2_. As schematically illustrated in Figure 1, a kind of small Pd nanoparticles with 30 nm hydrated size were used to accumulate in the liver in a targeting way post intravenous injection (Step 1), then rapidly capture the liver-passing H_2_ during hydrogen gas inhalation and store hydrogen in a solid form of Pd hydride (PdH) (Step 2), and finally catalyze the hydrogenation of ·OH for local and high-efficacy anti-CLD (Step 3). The local capture and catalytic hydrogenation characteristics of Pd nanoparticles enabled higher efficiency of ·OH elimination, remarkably enhancing the efficacy of hydrogen gas inhalation therapy in the mouse NASH models for CLD prevention and treatment. Such a local capture and catalytic strategy opens a new avenue for the targeted treatment of CLD.

## Results and Discussion

### Hydrogen capture and catalytic hydrogenation profiles of Pd nanoparticles

**Figure 2:**
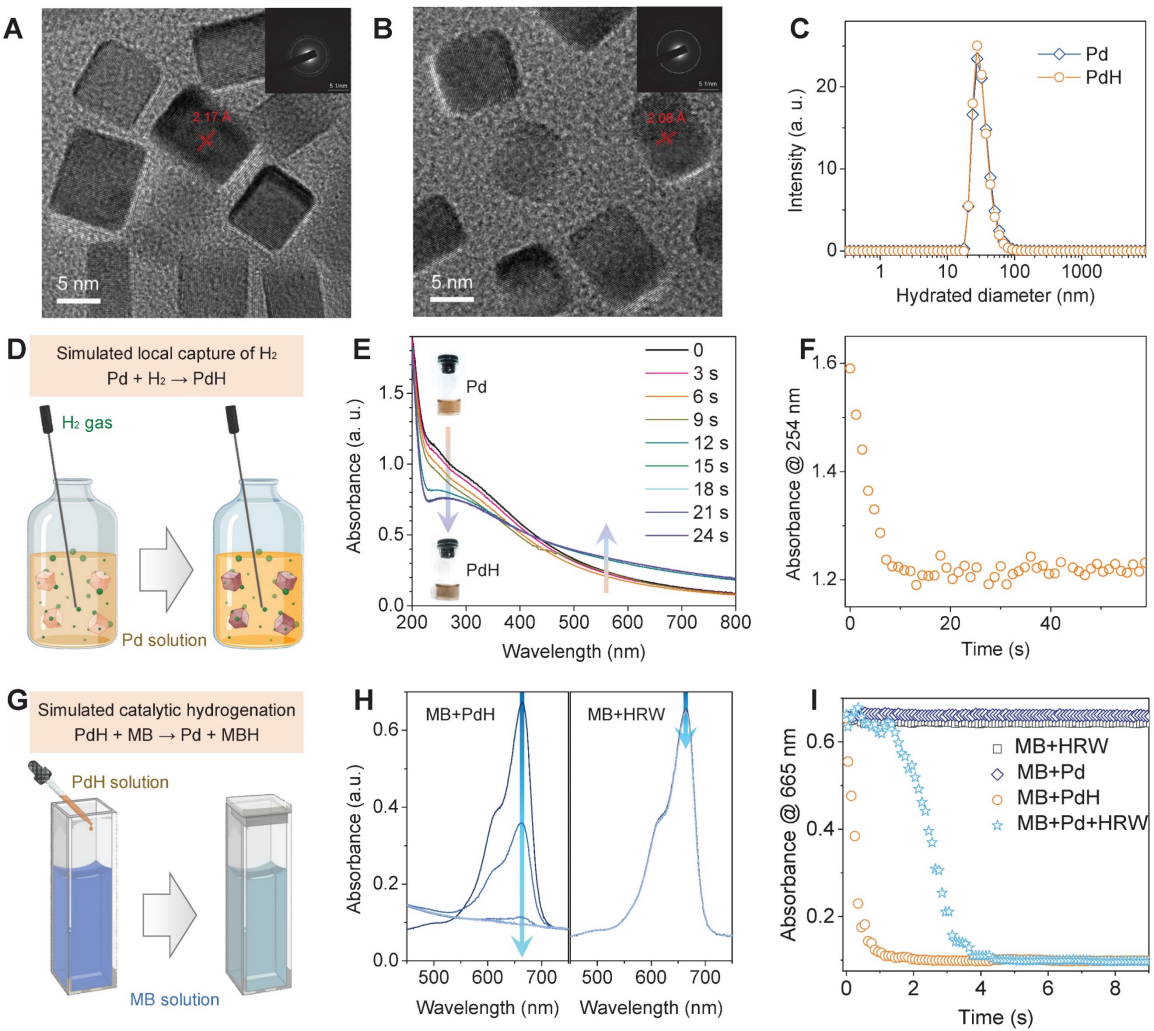
Characterization of dimension, hydrogen capture and catalytic hydrogenation features of Pd nanoparticles. HR-TEM images of PdH (**A**) and Pd nanoparticles (**B**) (insert images are the corresponding selected area electron diffraction patterns), their hydrated diameter distributions (**C**), simulated local hydrogen capture illustration (**D**) and corresponding UV absorption evolution (**E**) for monitoring dynamic process (**F**), and simulated catalytic hydrogenation illustration (**G**) and corresponding UV absorption evolution (**H**) for monitoring dynamic process (**I**).

Pd nanoparticles have been proven of high capability of hydrogen storage and catalytic hydrogenation 22,36-39, which is highly qualified to our therapeutic concept, and therefore were selected as a model nanomedicine to use in this work. For effective accumulation in liver, small size of Pd nanoparticles were synthesized by a facile method 22,36. TEM and DLS results indicated that the synthesized Pd nanoparticles had a cubic morphology, about 30 nm of hydrated diameter and high aqueous dispersion (Figure 2A‒C and Figure S1) in favor of liver targeted accumulation. The increase of crystalline plane spacing was attributed to hydrogen incorporation, leading to the worse crystallinity (Figure S2A). Local hydrogen capture of Pd nanoparticles was simulated by bubbling hydrogen gas into the aqueous solution of Pd nanoparticles, as illustrated in Figure 2D. The hydrogenation of Pd nanoparticles into PdH was monitored by the UV spectra (Figure 2E) 22. The decrease in the absorbance at 254 nm was used to reflect the dynamic process of hydrogen capture of Pd nanoparticles. From Figure 2F, it can be found that hydrogen molecules were rapidly captured by Pd nanoparticles at a second scale to form PdH nanoparticles with deeper color (the inset in Figure 2E) and weakened crystallinity (Figure S2) but without much change in morphology and size (Figure 2B-C and Figure S1).

The capability of catalytic hydrogenation of PdH nanoparticles was further investigated by using methylene blue (MB) as both a model molecule of free radicals and a color indicator of hydrogenation (Figure 2G). When HRW or PdH solution was injected into MB solution, the UV spectra were recorded to monitor the hydrogenation/reduction of MB in real time (Figure 2H), and the decrease in the absorbance at 665 nm was used to reflect the dynamic process of PdH-catalytic hydrogenation of MB (Figure 2I). It was clearly visible that MB was quickly reduced by PdH within 1 s rather than by HRW and Pd, indicating high capability of Pd/PdH nanoparticles for catalytic hydrogenation as expected.

Furthermore, we checked the local hydrogen capture and catalytic ·OH hydrogenation behaviors of Pd nanoparticles *in vitro*. An *in vitro* CLD model was built by treating mouse hepatocyte cell line AML-12 with 0.5 mM palmitic acid (PA). From Figure S3A-D, the incubation of AML-12 cells in a hydrogen incubator (50% H_2_) can scavenge the PA-induced ·OH, and Pd nanoparticles further enhanced the ·OH and ROS scavenging effect of hydrogen molecules, suggesting that intracellular Pd nanoparticles can capture hydrogen molecules in the culture medium and catalyze the hydrogenation of intracellular ·OH into H_2_O. As a result, Pd nanoparticles well assisted hydrogen molecules to inhibit the PA-induced lipid accumulation in AML-12 cells (Figure S3E-F). In addition, Pd nanoparticles were found to have the peroxidase/catalase-like catalytic activity in the tumor acidic microenvironment 40, but the CAT-like and POD-like behaviors of Pd nanoparticles could be not obvious due to a nearly neutral microenvironment in the NASH model and can be shielded by Pd-catalytic hydrogenation of ·OH.

### Proof of the therapeutic concept in the NASH model

**Figure 3:**
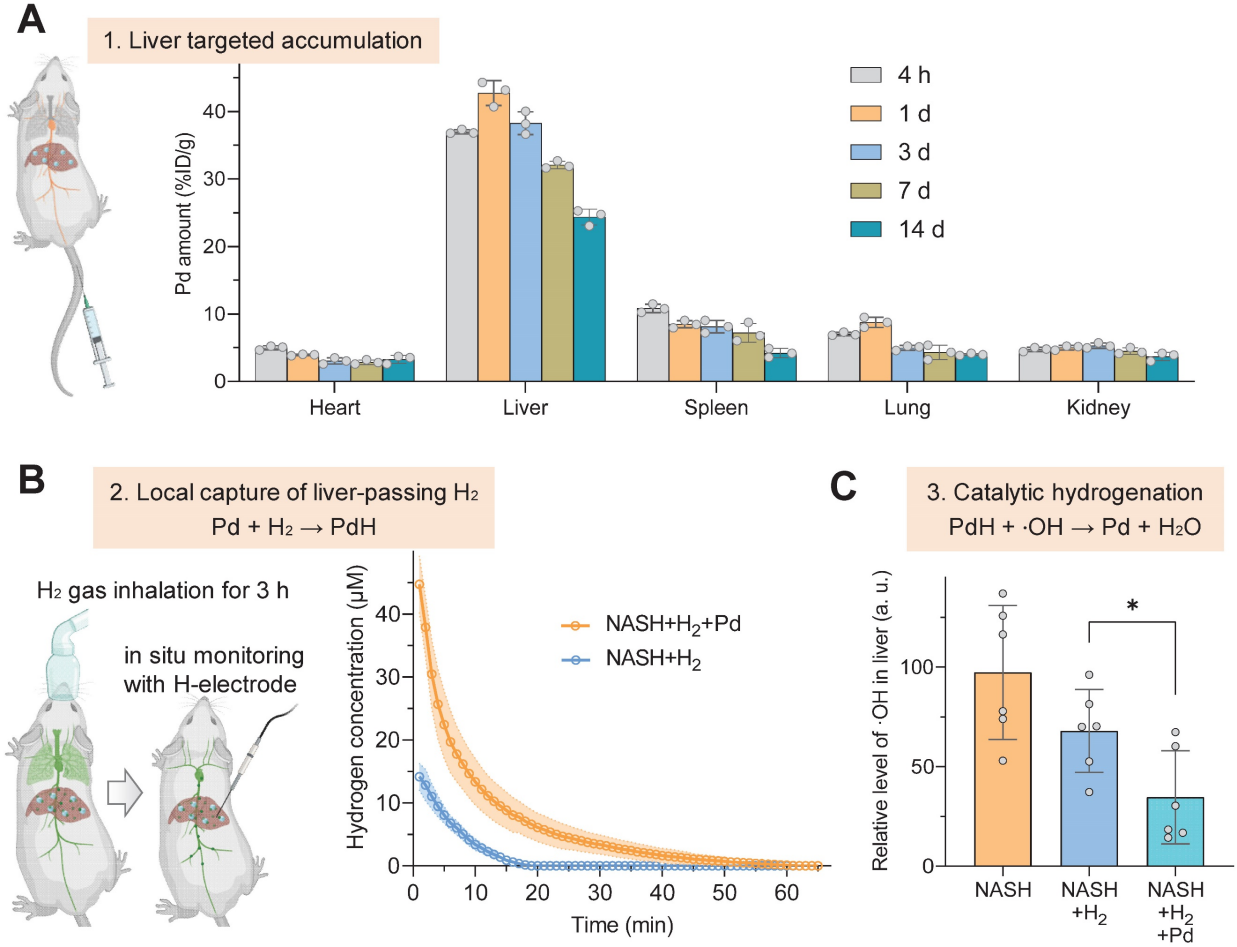
Liver-targeted accumulation, local hydrogen capture and catalytic hydrogenation behaviors of Pd nanoparticles in the NASH model. Biodistribution and intra-liver retention of Pd nanoparticles after intravenous injection (**A**), real-time *in situ* monitoring of hydrogen concentration in liver after Pd injection and 3-h hydrogen inhalation in the NASH mouse by a hydrogen electrode (*n* = 6) (**B**), catalytic hydrogenation capability of Pd nanoparticles for scavenging ·OH in the NASH mouse liver (**C**). Data were presented as mean ± standard deviation (Mean ± SD) with individual data. Student *t*-test was applied for comparison between groups. Difference was considered significant using asterisk as follows: *p < 0.05.

The therapeutic concept was further confirmed in the NASH mouse model from three aspects as illustrated in Figure 1. Firstly, the liver-targeted delivery of Pd nanoparticles was investigated. From the biodistribution in Figure 3A, a major proportion of Pd nanoparticles retained in liver after intravenous injection. We did not observe a distinct change in the particle size of Pd nanoparticles in serum for 4 hours (Figure S2B), indicating good stability in serum. From pharmacokinetic curve (Figure S4), Pd nanoparticles were gradually cleared from blood. The accumulation amount of Pd in liver reached a peak (42.7% ID/g) at 24 h after injection and gradually declined with time, implying that Pd nanoparticles can be gradually excreted from liver. Fourteen days later, 24.4% ID/g of Pd particles still preserved in liver, which ensured a long-term Pd-mediated catalytic treatment of chronic liver diseases (Figure 3A). TEM analysis of liver extracted at 7 d after injection showed that Pd nanoparticles aggregated in lysosome and endosome in Kupffer cells in liver, and also adhered onto both mitochondria and endoplasmic reticulum in hepatocytes in favor of Pd locally scavenging ·OH in various cells in liver (Figure S5). Hepatic macrophages play an important role in NASH development and progression. Liver-resident Kupffer cells recruit peripheral monocytes to the liver and rapidly differentiate them into proinflammatory macrophages which eventually makes contribution to NASH fibrosis. The accumulation of Pd nanoparticles in hepatic macrophages will provide opportunity for local anti-inflammatory treatment in this work.

Secondly, the *in vivo* local hydrogen capture behavior of Pd nanoparticles was investigated in a mouse NASH model. NASH mice were intravenously injected with a single dose of 10 mg/kg Pd nanoparticles, then inhaled 4% hydrogen gas for 3 h, and immediately anesthetized followed by real-time *in situ* monitoring of hydrogen concentration in liver using a hydrogen electrode, as demonstrated in Figure 3B. The data at the onset time point in Figure 3B indicated that Pd nanoparticles indeed captured a large amount of H_2_ in liver, which was three-fold higher than the case without Pd nanoparticles, and stored hydrogen in the form of PdH (Figure 2D‒F). Moreover, hydrogen concentration in the liver of mice which only inhaled hydrogen gas quickly declined to zero after 20 min, owing to high dispersion of hydrogen molecules 29. By comparison, Pd nanoparticles enabled prolonged hydrogen dispersion, because of lower mobility of PdH nanoparticles in the body compared with hydrogen molecules.

Thirdly, the *in vivo* catalytic hydrogenation behavior of Pd nanoparticles for scavenging ·OH was further investigated in the mouse NASH model. After Pd injection and 3-h hydrogen gas inhalation, the liver was extracted and the contents of ·OH and reactive oxygen species (ROS) in liver were detected. In comparison with only hydrogen gas inhalation, the combination of Pd nanoparticles injection with hydrogen gas inhalation more significantly lowered the ·OH level in the NASH liver (*p* < 0.05) (Figure 3C) so that the ROS concentration in the NASH liver can recover to the normal level (Figure S6), indicating high capability of injected Pd nanoparticles for local catalytic ·OH hydrogenation/scavenging.

### Therapeutic outcomes of mild NASH prevention

Successful proof of our therapeutic concept encouraged us to explore the therapeutic benefits of CLD prevention and treatment from our therapeutic strategy. The mild and moderate NASH models as two representative examples of CLD were built to investigate therapeutic outcomes 38. In order to harvest the maximum outcomes of anti-NASH, PdH nanoparticles were injected in the following therapeutic experiments to achieve the first elimination of ·OH since its oxidation product Pd can still play a role of catalytic hydrogenation.

**Figure 4:**
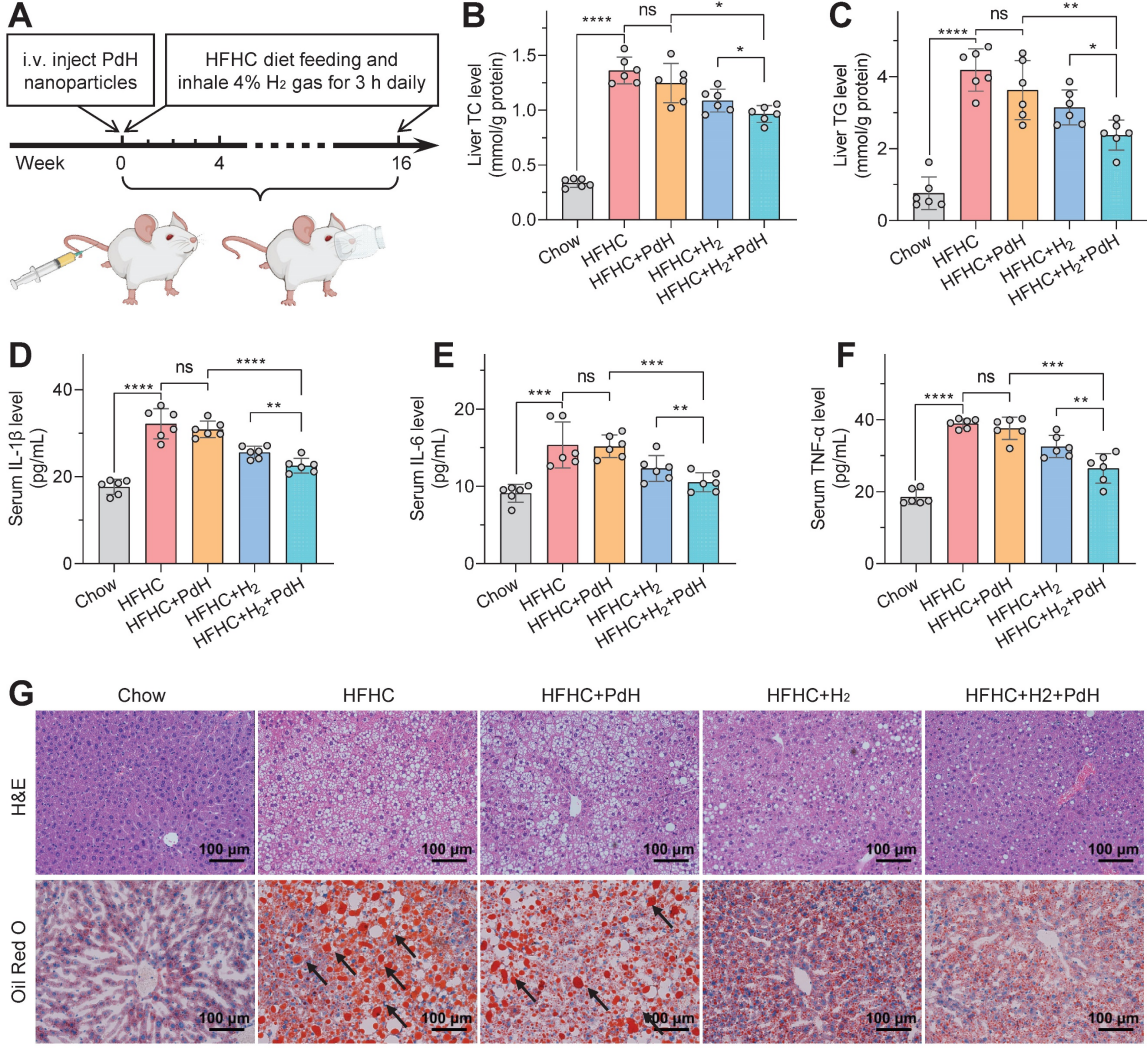
The therapeutic performances of PdH injection plus hydrogen inhalation for preventing mild NASH. Therapeutic procedure of mild NASH prevention (**A**), total cholesterol (**B**) and triglyceride (**C**) contents in livers, systemic inflammation levels involving IL-1β (**D**), IL-6 (**E**) and TNF-α (**F**), and H&E and Oil Red O staining (black arrow towards red spots representing neutral triglycerides and lipids) images of liver sections at the end of treatment (**G**). All the data were presented as mean ± standard deviation (Mean ± SD) with individual data. Student *t*-test was applied for comparison between groups. Difference was considered significant using asterisk as follows: *p < 0.05, **p < 0.01, ***p < 0.001, and ****p < 0.0001.

As for the NASH prevention, the mild NASH model was built by daily feeding C57BL/6J mice with a high-fat/high-cholesterol (HFHC) diet, and intravenously injected with PdH nanoparticles at a dosage of 10 mg/kg at the beginning of the model building, and then daily inhaled 4% hydrogen gas for 3 h throughout the whole prevention period (16 weeks), as demonstrated in Figure 4A. From Figure 4 and Figure S7‒S15, the 16-week HFHC diet induced significantly higher liver/body weights (Figure S7), higher liver/serum glucose/lipid levels (Figure 4B,C,G and Figure S8‒S12) involving total cholesterol (TC), triglyceride (TG), non-high density lipoprotein cholesterol (non-HDL-C) and non-esterified fatty acid (NEFA), higher liver enzyme levels (Figure S13) involving alanine aminotransferase (ALT) and aspartate aminotransferase (AST), and higher hepatic inflammation levels (Figure 4D‒F, Figure S14 and Figure S15) compared to the blank control group (Chow) with chow diet, suggesting successful building of the mild NASH model. Individual daily hydrogen inhalation ameliorated these representative parameters of NASH to a limited extent, while the influence of individual one-off injection of PdH nanoparticles was almost negligible. By comparison, the proposed therapy with PdH injection plus daily hydrogen inhalation exhibited a significantly higher improvement in both systemic inflammation (Figure 4D‒F, Figure S14 and Figure S15) and lipid metabolic dysfunction (Figure 4B, C, G and Figure S7‒S12), which should be attributed to the hydrogen capture and catalytic hydrogenation effects of Pd nanoparticles.

As regard to systemic inflammation, the therapy with PdH injection plus daily hydrogen inhalation more remarkably declined the levels of various typical serum inflammatory cytokines including IL-1β, TNF-α and IL-6 compared to other treatment groups (Figure 4D‒F), which could be due to that the hepatic oxidative stress had been completely eradicated, as indicated by the attenuation of the ·OH and ROS levels in the mild NASH liver to the normal (Figure S14). It resulted in the recovery of the endogenous anti-oxidative capability of liver approximately to the normal level, as suggested by the enhancement of superoxide dismutase (SOD) and glutathione (GSH) levels (Figure S15). In brief, the hydrogen capture and catalytic hydrogenation effects of Pd nanoparticles greatly enhanced the anti-inflammation outcomes of daily hydrogen gas inhalation.

As the result of systemic anti-inflammation, the therapy with PdH injection plus daily hydrogen inhalation more remarkably improved hepatic lipid metabolism than other treatment groups with individual PdH injection or with individual hydrogen gas inhalation (Figure 4B, C, G and Figure S7‒S12). The glucose/lipid levels in both liver and serum were considerably depressed (Figure 4B, C, G and Figure S8‒S12), leading to the complete recovery of liver and body weights amazingly (Figure S7) and the glucose-feedback decrease in the insulin level (Figure S9B) 41. Moreover, the improvement of lipid accumulation in liver was directly observed by HE and Oil Red O imaging of liver sections (Figure 4G) and by B-mode non-invasive ultrasound (US) imaging (Figure S12). Furthermore, the significant decrease of ALT and AST levels implied the recovery of liver functions to a certain extent (Figure S13), as inflected by augmented hepatic capability in glucose tolerance (Figure S10) and insulin tolerance (Figure S11). The above results consistently concluded that the proposed therapeutic concept of local hydrogen capture and catalytic ·OH hydrogenation brought additional benefits for hydrogen gas inhalation therapy, efficiently preventing the progression of the mild NASH.

### Therapeutic outcomes of moderate NASH treatment

**Figure 5:**
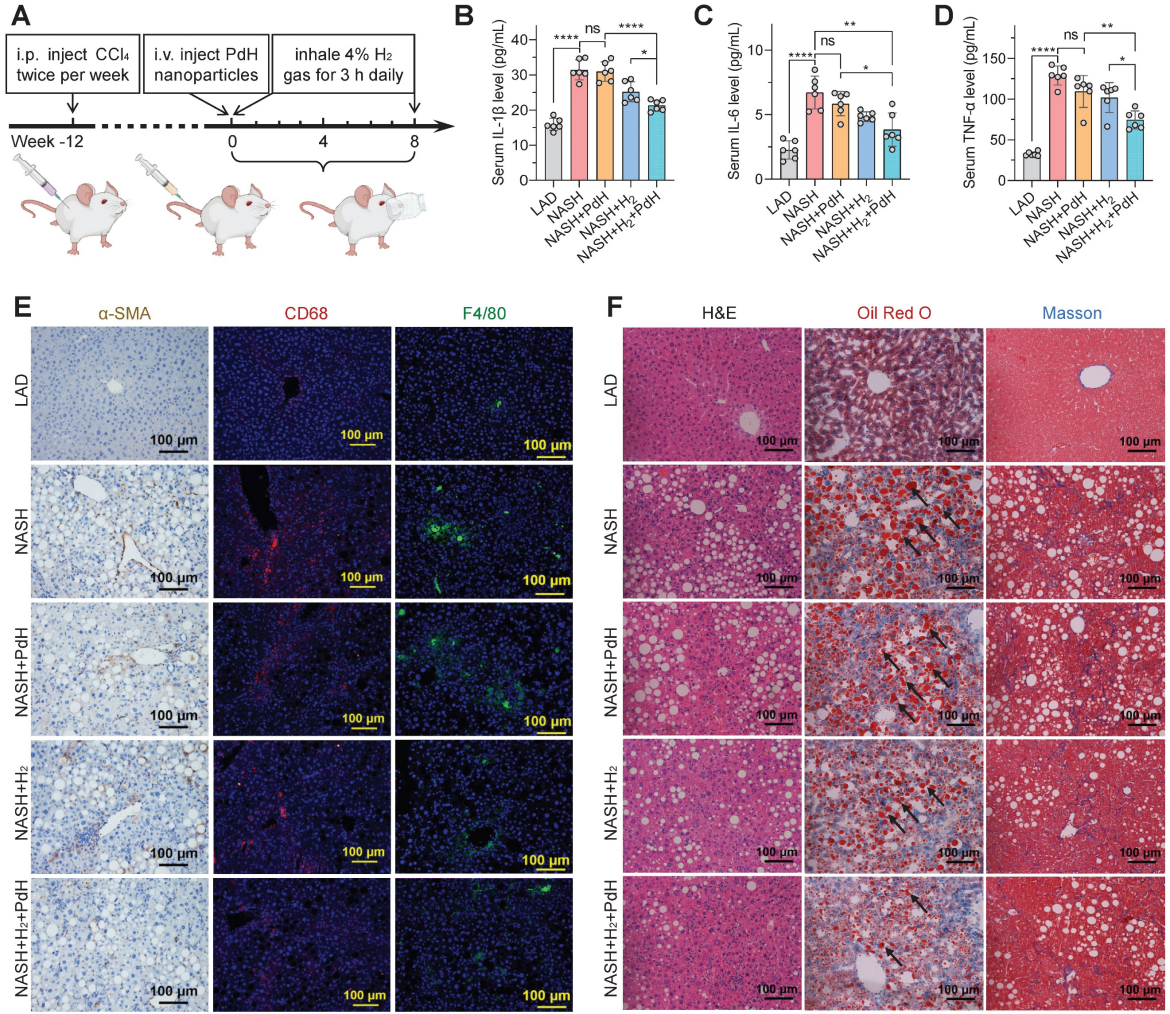
The therapeutic performances of PdH injection plus hydrogen inhalation for alleviating moderate NASH. Model building and therapeutic procedures (**A**), the levels of serum cytokines including IL-1β (**B**), IL-6 (**C**), TNF-α (**D**), IHC and immunofluorescence detection of α-SMA, CD68 and F4/80 levels in liver sections (**E**), and the H&E, Oil Red O (black arrow towards red spots representing neutral triglycerides and lipids) and Masson's trichrome staining (blue representing collagen) images of liver sections at the end of treatment (**F**). All the data were presented as mean ± standard deviation (Mean ± SD) with individual data. Student *t*-test was applied for comparison between groups. Difference was considered significant using asterisk as follows: *p < 0.05, **p < 0.01, ***p < 0.001, and ****p < 0.0001.

Further, the moderate NASH model with a higher malignance than the mild one was used to explore the performances of our therapeutic concept. The moderate NASH model was firstly established by daily feeding C57BL/6J mice with a western diet and intraperitoneally injecting 0.2 μL/g CCl_4_ twice per week for the whole period (20 weeks), and the chow diet mice without CCl_4_ injection were used as a normal control group (LAD) 41,42. The treatment procedure of the moderate NASH model was similar to that of the mild one. As demonstrated in Figure 5A, the NASH mice were injected with PdH nanoparticles at a safe dosage of 10 mg/kg at week 12 22, and then daily inhaled 4% hydrogen gas which is a widely accepted concentration for hydrogen inhalation exhibiting biomedical effect and is at the lower flammability limit for 3 h throughout the whole treatment period (8 weeks).

Compared with the above-mentioned mild NASH model, the moderate one indeed exhibited higher malignance in both the systemic inflammation (Figure 5B‒E) and hepatic fibrosis levels (Figure 5F) and liver weight/functions (Figure S16‒S17). Similar to the results of mild NASH prevention, individual PdH injection did not exhibit significant change in these representative parameters in the moderate NASH treatment, while individual hydrogen gas inhalation caused significant but weak therapeutic efficacies, but the combination of PdH injection and hydrogen gas inhalation resulted in the best therapeutic outcomes. It suggested that the proposed therapeutic concept of local hydrogen capture and catalytic ·OH hydrogenation can also augment the efficacy of hydrogen gas inhalation therapy of the moderate NASH, exhibiting the great strength of the proposed therapeutic strategy.

Noticeably, even though the moderate NASH had higher systemic inflammation than the mild one, the therapy with PdH injection and daily hydrogen gas inhalation can still efficiently scavenge both local and systemic inflammations, as indicated by the visible attenuation of circulating IL-1β, IL-6 and TNF-α levels (Figure 5B‒D) and the remarkable depression of activated hepatic stellate cells (α-SMA) 43 and pro-inflammatory macrophages (CD68) (Figure 5E). The liver-retaining PdH nanoparticles seemed like a filter of inflammation which can locally capture the liver-passing hydrogen molecules and continuously eliminate the liver-passing ·OH (Figure 1). In addition, the decrease in the liver MDA level and the increase in the liver GSH level reflected the enhancement in the anti-oxidative capability of liver (Figure S18).

Corresponding to the decrease in the hepatic and systemic levels of glucose, non-HDL-C and NEFA (Figure S19‒S22), hepatic lipid accumulation was much attenuated by the therapy with PdH injection and daily hydrogen gas inhalation according to the results from HE and Oil Red O imaging (Figure 5F), related histology scoring (Table S1) and US imaging (Figure S23), displaying the decrease of the ratio of liver weight to body weight (Figure S16). The insignificant effect either by hydrogen inhalation or by Pd injection in GTT assay was possibly attributed to the NASH model we applied. Insulin resistance is at an intermediate level when CCl_4_ is added to Western diet so that the slight improvement of insulin resistance seems to be unnoticeable 42. It is worth noting that accompanied by serious inflammation and hepatic lipid metabolic dysfunction, liver fibrosis was clearly visible in the moderate NASH mice and can also be significantly attenuated by the combined therapy (Figure 5F). These results indicated that our therapeutic strategy did not only improve the outcomes of hydrogen therapy but also effectively blocked the evolution of the moderate NASH.

**Figure 6:**
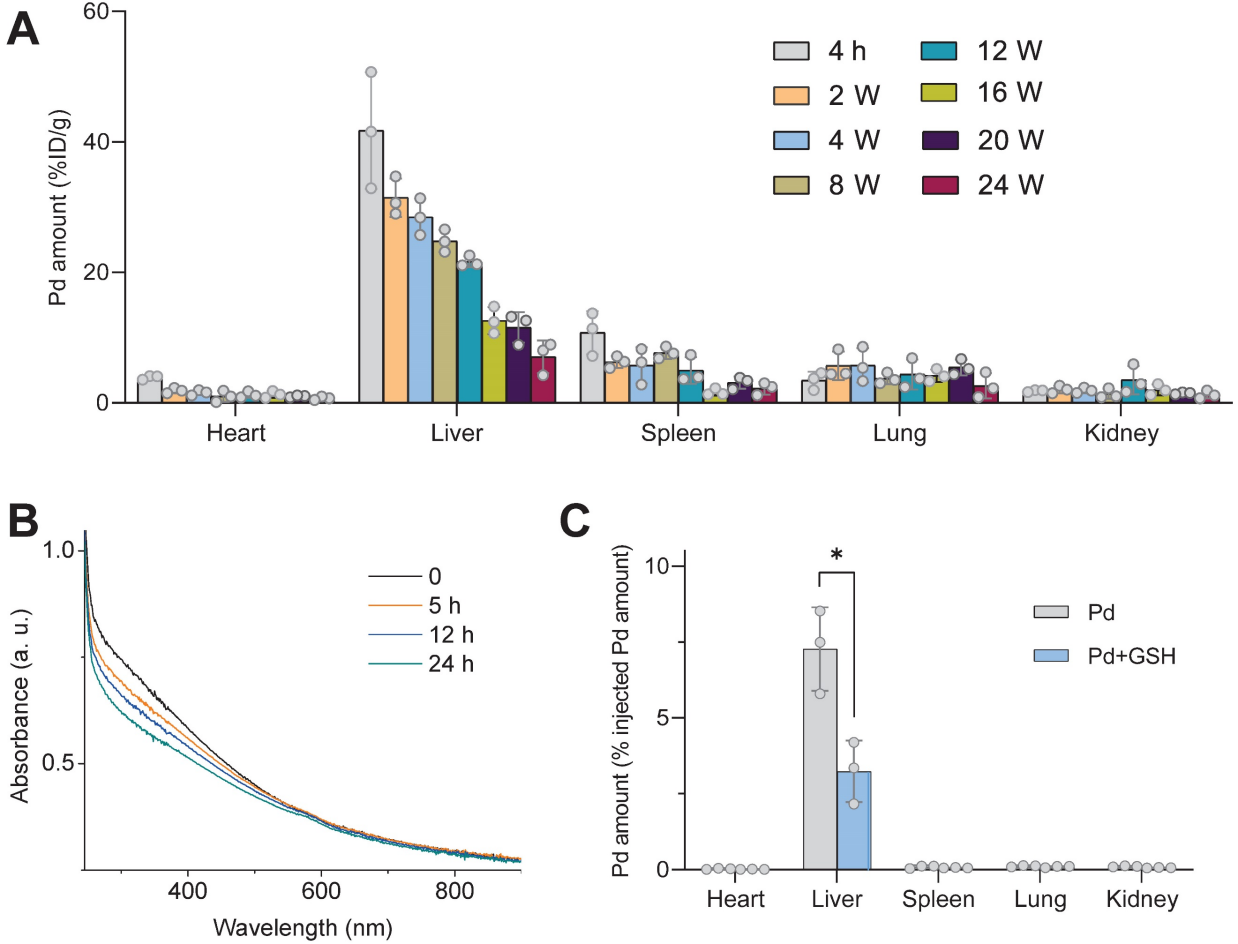
The GSH-accelerated excretion of Pd from the body of mice. The biodistribution of Pd in main organs after intravenous injection of PdH for different time durations (**A**), the change in the absorbance of the aqueous mixed solution of Pd (20 μg/mL) and GSH (10 mM) with time (**B**), and the biodistribution of Pd 12 weeks after intravenous Pd injection without (Pd group) and with everyday intramuscular injection of 400 mg/kg/d GSH (Pd+GSH group) after 8 weeks (**C**). Data were presented as mean ± standard deviation (Mean ± SD) with individual data. Student *t*-test was applied for comparison between groups. Difference was considered significant using asterisk as follows: *p < 0.05.

Furthermore, the excretion behavior of Pd from the body of mice was investigated. From Figure 6A, Pd can gradually be excreted from the liver, and after intravenous injection of PdH nanoparticles for 24 weeks, about a half of Pd had been excreted from the liver. No toxicity was observed based on H&E staining of liver, lung, spleen and kidney (Figure S24). In order to accelerate the Pd excretion after the end of treatment (8 weeks) to avoid the potential risk of biosafety, we tried to inject a chelator to decompose Pd in the liver. In our previous report, we found that Pd nanoparticles can be easily decomposed by sulfourea, owing to strong coordination between them 44, and GSH is a potent chelator which facilitates intracellular transport and excretion of heavy metals 45,46. Therefore, we hypothesized that GSH with intense ability of coordination with Pd could decompose Pd nanoparticles and accelerate their removal from the body. As expected, we found that GSH was indeed able to decompose Pd nanoparticles in the aqueous solution as their characteristic absorption became weak with the increase of mixture time (Figure 6B). Therefore, we intramuscularly inject GSH (400 mg/kg/d) every day 8 weeks after PdH injection. At week 12, it can be clearly found that the Pd excretion rate of liver increased by about one-fold, and the residual amount of Pd in the liver reduced to only about 3% (Figure 6C). It can be expected that Pd can be mostly eliminated under the assistance of GSH.

### The molecular mechanism for enhanced hydrogen therapy of NASH

**Figure 7:**
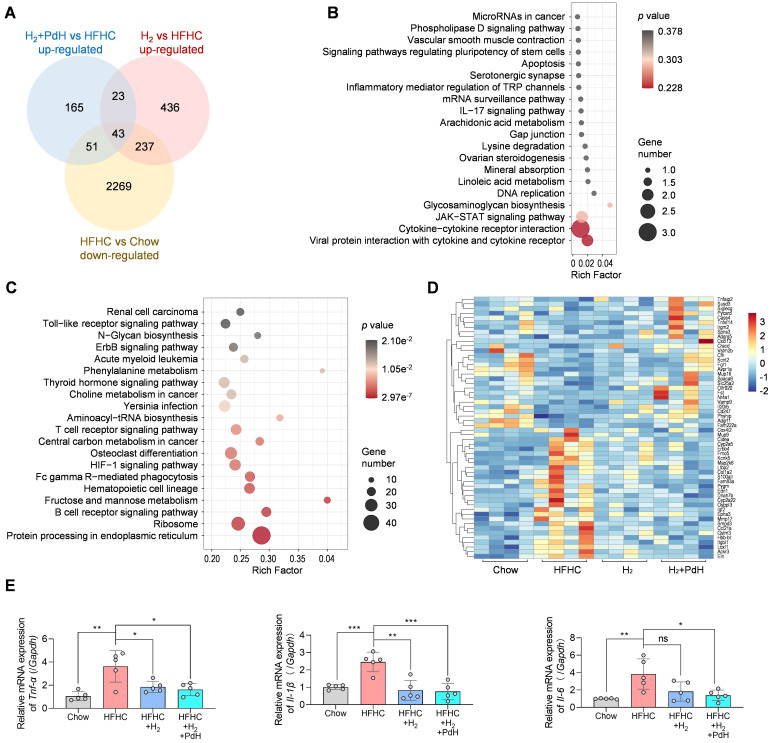
Transcriptome analysis of liver samples. The Venn diagram of the DEGs in various comparisons (**A**), the KEGG enrichment analysis of the 43 genes clustered in the Venn diagram (**B**), the KEGG enrichment analysis of all the DEGs in comparison of H_2_+PdH and H_2_ groups (Log_2_|fold change| > 0, *p* value < 0.05) (**C**), the heatmap of DEGs in enriched pathways in comparison of H_2_+PdH and H_2_ groups (**D**), mRNA expression levels of TNF-α, IL-6 and IL-1β in liver tissues examined by real-time qPCR (**E**). Data were presented as mean ± standard deviation (Mean ± SD) with individual data. Student *t*-test was applied for comparison between groups. Difference was considered significant using asterisk as follows: *p < 0.05, **p < 0.01, ***p < 0.001.

To explore the underlying molecular mechanism for hydrogen therapy of NASH, we performed a transcriptome analysis of liver samples. A total of 16143 mRNAs were identified and then the enrichment analysis of differentially expressed genes (DEGs) was performed. As demonstrated by the Venn diagram in Figure 7A, 2600 DEGs were down-regulated in the mild NASH model group (HFHC) compared with the normal control group (Chow) (log_2_(fold change) < 0, *p* value ≤ 0.5), and 282 and 739 DEGs were up-regulated in the H_2_ and H_2_+PdH groups of treatment compared with the model control group (HFHC), respectively (log_2_(fold change) > 0, *p* value ≤ 0.5). It can be found that 43 genes which were shared in these three clusters were enriched in the KEGG pathways mainly involving cytokine-cytokine receptor interaction, Janus kinase (JAK)-signal transducer and activator of transcription (STAT) signaling pathway and viral protein interaction with cytokine and cytokine receptor, *etc.* (Figure 7A-B) suggesting that H_2_ reversed the inflammatory responses of immune system in accordance with the above immunohistochemical results (Figure 5E).

By comparing the DEGs between H_2_+PdH and H_2_ groups, the most influenced KEGG pathways mainly included protein processing in endoplasmic reticulum, ribosome, hematopoietic cell lineage, central carbon metabolism, fructose and mannose metabolism, B cell receptor signaling pathway, and T cell receptor signaling pathway (log_2_|fold change|>0, *p* value < 0.5, Figure 7C). It indicated that compared with individual hydrogen gas inhalation, our therapeutic strategy with the combination of PdH injection with hydrogen gas inhalation further impacted a wide range of both metabolic and anti-inflammatory immune pathways, well explaining the above-verified enhanced NASH improvement in liver lipid metabolism and local and systemic inflammation. Furthermore, from the corresponding heatmap in Fig 7D, compared to individual hydrogen gas inhalation, the therapy with the combination of PdH injection with hydrogen gas inhalation further enhanced the up-regulation of Nr4a1 and the down-regulation of Cox4i2, Ccl21a and Itgbl1. Transcriptional levels of TNF-α, IL-6 and IL-1β detected by real-time qPCR of liver tissues confirmed that compared to individual hydrogen gas inhalation, our strategy further inhibited the expression of liver IL-6 and IL-1β (Figure 7E).

**Figure 8:**
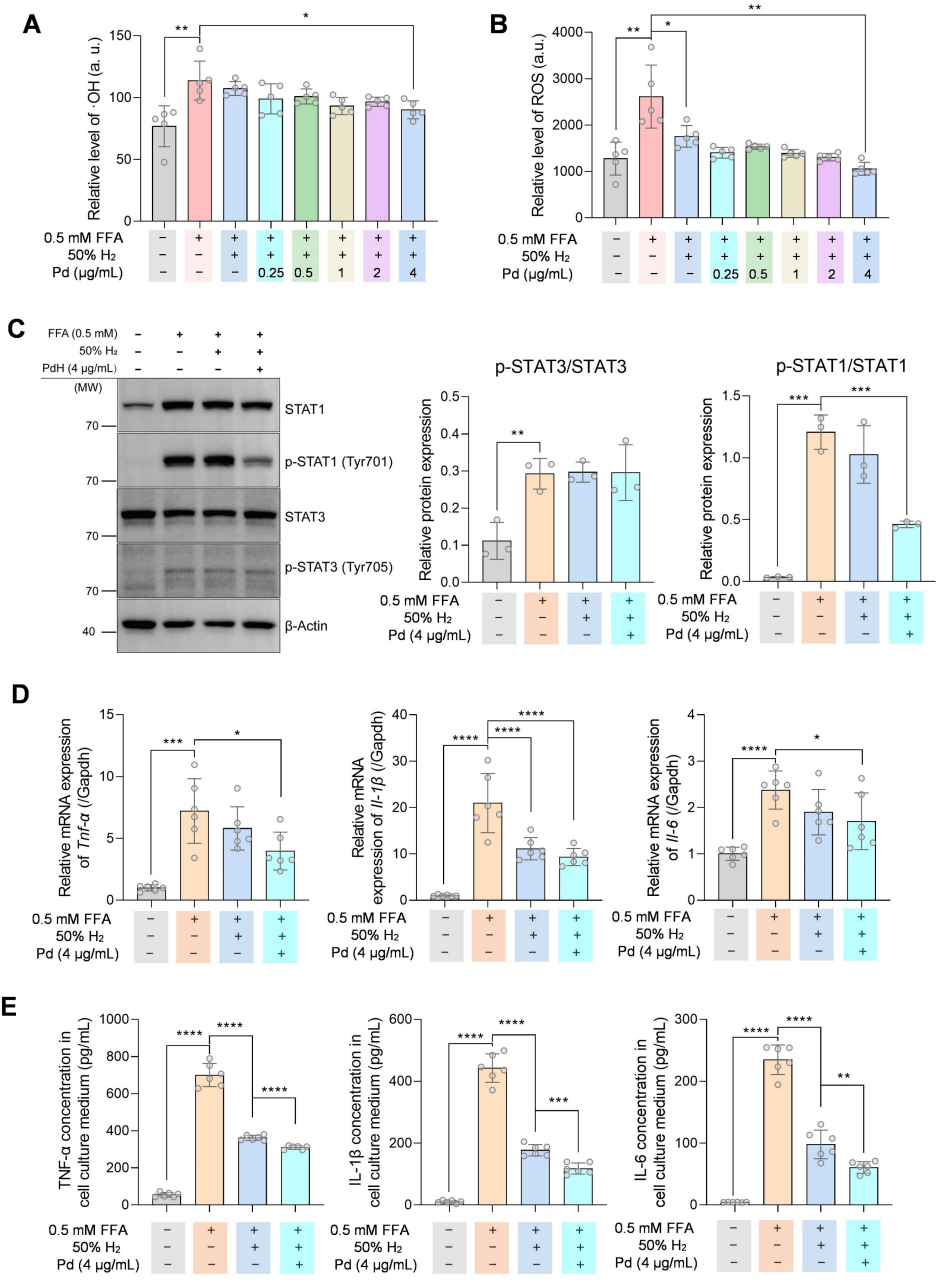
The anti-inflammatory effect and mechanism of PdH nanoparticles in THP-1 derived macrophages. Intracellular ·OH (**A**) and ROS (**B**) levels in 0.5 mM FFA-treated THP-1 cells incubated with different concentrations of PdH. Western blot analysis of STAT1, phosphor-STAT1, STAT3 and phosph-STAT3 of 4 μg/mL PdH-incubated and 0.5 mM FFA-treated THP-1 cells (**C**), real-time qPCR analysis (**D**) and quantification of cytokines in the THP-1 cell culture medium (**E**) of TNF-α, IL-1β and IL-6. All the data were presented as mean ± standard deviation (Mean ± SD) with individual data. Student *t*-test was applied for comparison between groups. Difference was considered significant using asterisk as follows: *p < 0.05, **p < 0.01, ***p < 0.001, and ****p < 0.0001.

To evaluate the anti-inflammation ability of PdH nanoparticles *in vitro*, we incubated THP-1 derived macrophages with 0.5 mM free fatty acid (FFA) to mimic the liver macrophages in the NASH condition. From Figure 8 A-B, intracellular ·OH and ROS levels in THP-1 cells decreased on a PdH dose-dependent manner when the combination of PdH administration with hydrogen incubation was treated. When 4 μg/mL PdH was applied to THP-1 cells, the oxidative species were significantly lower compared with the case of individual hydrogen incubation (Figure 8 A-B). Western blot analysis demonstrated that though the STAT3 activity was unchanged when hydrogen and/or PdH was applied, the activity of STAT1 was significantly inhibited in PdH+H_2_-treated cells (Figure 8C), resulting the decreased transcriptional levels and secretion of TNF-α, IL-1β, and IL-6 in FFA-induced THP-1 derived macrophages (Figure 8 D-E). All these data indicated an additional benefit of anti-NASH from alleviated inflammation, balanced glucose homeostasis and improved lipid metabolism 47-49.

## Conclusion

In summary, this work proposed the new therapeutic concept of local hydrogen capture and catalytic hydrogenation for anti-CLD, and successfully verified the concept by the combination of PdH nanoparticles injection with hydrogen gas inhalation *in vitro* and *in vivo*. In the mild and moderate NASH models, we confirmed that our therapeutic strategy remarkably enhanced the outcomes of hydrogen gas inhalation by improving liver lipid metabolism and local/systemic inflammation. On account of the biosafety of molecular hydrogen and Pd nanoparticles, the cost-effective benefit of combining single dose injection of PdH with hydrogen inhalation, and liver-targeted effect, the proposed therapeutic concept is a promising strategy for clinical prevention and treatment of CLD.

## Methods

### Synthesis and characterization of Pd nanoparticles

Pd nanoparticles were synthesized by a redox method 22,36. An aqueous solution (11 mL) of PVP (106.4 mg), AA (60 mg), KBr (301 mg) and Na_2_PdCl_4_ (56.3 mg) were prepared and then heated for 3 h at 80 °C under magnetic stirring. The solution was cooled to room temperature and then as-synthesized Pd nanoparticles were collected and purified using hyper-filtration tubes (MW cut-off 100 kDa, Millipore) by 30 min of centrifugation at 1500×*g* and 3-time washing using deionized water. Finally, Pd nanoparticles were re-dispersed in deionized water and stored in dark until use. The morphology and size of Pd and PdH nanoparticles were characterized by TEM (JEM-2100F). The hydrated size of nanoparticles was measured on a Malvern Zetasizer Nano ZS90. XRD patterns were collected on a M21X diffractometer (Cu Kα, λ = 1.54056 Å).

### Simulation of hydrogen capture and catalytic hydrogenation *in vitro*

The hydrogen capture of Pd nanoparticles simulated in the solution was monitored by UV technique. The aqueous solution of Pd nanoparticles (3 mL, 25 μg/mL) was bubbled with pure hydrogen gas which was generated by a hydrogen generator. During the process, the UV spectra were recorded in time. After hydrogen gas bubbling for 1 h, PdH nanoparticles were collected and re-dispersed in water for the following immediate use.

Methylene blue (MB) was used as a probe to detect the catalytic hydrogenation behavior of Pd nanoparticles. Firstly, the concentrated aqueous solution of PdH nanoparticles (1 mL, 25 μg/mL) was quickly injected into the aqueous solution of MB (2 mL, 24 μg/mL), and meanwhile the UV spectra were collected in time. As to the Pd and HRW controls, the aqueous solution of Pd nanoparticles or HRW at the equal concentration was quickly injected into the aqueous solution of MB aqueous followed by UV monitoring.

Intracellular hydrogen capture and catalytic hydrogenation were testified with a PA-induced hepatic cell model of NASH. Mouse hepatocyte AML-12 cell line obtained from Shanghai Institutes for Biological Science, CAS was maintained in a complete AML-12 cell medium (Procell, China, CM-0317). Cells were seeded in black 96-well microtiter plates at a density of 4×10^5^/mL and cultured overnight in a normal cell incubator (5% CO_2_, 21% O_2_, and 74% N_2_). Palmitate acid (PA) and Pd nanoparticles were added into the cells and incubated for 12 h in a hydrogen incubator (Wuxi Puhe, PH-A2) which provided a 5% CO_2_, 21% O_2_, 50% H_2_ and 24% N_2_ environment. Then cell plates were transferred into the normal cell incubator for another 10 h. Cell viability assay was performed using a CCK-8 kit (Dojindo, CK04). Intracellular ROS was measured using a reactive oxygen species assay kit (Solarbio, CA1410). The concentration of ·OH was measured using a 3ʹ-(*p*-hydroxyphenyl) fluorescein (HPF, 10 μM, Invitrogen™, H36004) and the fluorescence at 490/515 nm was detected on a microplate reader (Molecular Devices, Spectra Max M5).

THP-1 cells were maintained in RPMI-1640 medium supplemented with 10% FBS and were differentiated into macrophage-like cells by incubation with 100 nM Phorbol 12-myristate 13-acetate (PMA, Sigma-Aldrich, P8139) for 48 h. Pro-inflammatory phenotype was induced by incubation with 0.5 mM free fatty acid (FFA, oleic acid: palmitate acid = 2:1). Cells were incubated with FFA and PdH nanoparticles and maintained in hydrogen incubator for 12 h and then transferred to the normal cell incubator for 10 h.

### Intracellular lipid staining and quantification

AML-12 cells were fixed with 4% paraformaldehyde and stained with 1 μg/mL BODIPY (Invitrogen™, D3922) and 1 μg/mL DAPI (Sigma-Aldrich, D9542). Quantification was conducted on a microplate reader (Molecular Devices, SpectraMax M5) by detection of fluorescence at 485/525 nm (BODIPY) and 364/454 nm (DAPI). Images were obtained using a Lionheart FX Automated Microscope.

### Western blot

Total soluble protein of cell samples were obtained by lysis with RIPA buffer (Solarbio, R0010) supplemented with a protease inhibitor cocktail (cOmplete Ultra Tablets, Roche, 05892970001) and phosphatase inhibitor cocktail (Sigma-Aldrich, P0044). Protein concentrations were examined and equalized with a BCA kit (PC0020, Solarbio). Protein samples were applied to SDS-PAGE and then transferred to PVDF membranes (Millipore) by electroblotting. Immunoblotting was conducted using specific antibodies as follows: STAT1 (Proteintech, 10144-2-AP), p-STAT1(Tyr701) (CST, #9167), STAT3 (CST, #9139), p-STAT3(Tyr705) (CST, #9145), β-Actin (Proteintech, 66009-1-Ig). Images were taken on a chemiluminescence detection system (ChemiScope 6200Touch, Clinx Science Instruments, China) and analyzed using Fiji Image J 1.53e.

### Enzyme-linked immunosorbent assay (ELISA)

Cell culture mediums from each well in 6-well plates (2 mL) were concentrated with a vacuum centrifugal concentrator (Scientz-1LS) and resuspended with 400 μL PBS. The levels of TNF-α, IL-6, IL-1β in the resuspended solution were measured using human ELISA kits (ExCell Bio) according to the manufacturer's instructions.

### Real-time qPCR

Mouse liver tissues or THP-1 derived macrophages were treated in 1 mL TRIzol™ reagent (Invitrogen, 15596018) and then RNAs were isolated by phenol-chloroform extraction. Complementary DNA (cDNA) synthesis was performed using HiFi Script gDNA Removal RT MaterMix (CWBIO, China, CW2020M). Real-time PCR (qPCR) was conducted using TransStart Top Green qPCR SuperMix (Transgene Biotech, China, AQ131) on an Applied Biosystems™ Quant Studio 3 Real-time PCR system. Relative expressions of genes were calculated using the 2^-ΔΔ^ method normalized to *Gapdh*. Primers for qPCR were designed and synthesized at Shanghai Sangon Biothech (Table S2).

### Biodistribution and GSH-accelerated excretion of Pd nanoparticles

C57B/6J mice (8 weeks old, male) were intravenously injected with 250 μL PBS containing 10 mg/kg Pd nanoparticles. Mice were euthanatized, and their heart, liver, spleen, lung and kidney were dissected at 2 h, 4 w, 8 w, 12 w, 16 w, 20 w, and 24 w post injection (*n* = 3). Dissected organs were weighed and digested with aqua regia, heated to dryness and diluted to a certain volume with deionized water. The quantification of Pd amount in organs was performed using inductively coupled plasma-atomic emission spectrometry (ICP-AES, Agilent Technologies, USA).

To accelerate the excretion of Pd, GSH was used to decompose Pd nanoparticles. For *in vitro* study, UV spectra of aqueous solution of Pd nanoparticles (20 μg/L), Pd nanoparticles (20 μg/L) were dispersed in the aqueous solution of GSH (10 mM), whose UV spectra were monitored at different time points to determine the decomposition of Pd nanoparticles. For *in vivo* study, after C57B/6J mice were intravenously injected with Pd nanoparticles (10 mg/kg) for 8 weeks, daily intramuscular injection of 400 mg/kg reductive GSH was administrated. At week 12, mice were sacrificed, and their heart, liver, spleen, lung and kidney were dissected for Pd quantification by ICP analysis (*n* = 3). The mice administered with Pd nanoparticles and without GSH were used as the control.

### Protocols of building the mild and moderate NASH mouse models

C57BL/6J mice (Charles River Laboratories, Beijing, China) were maintained under a 12-h light/12-h dark cycle in an environment of 24±1°C and 55-60% humidity *ad libitum*. All the procedure of animal experiments was conducted in accordance with the guidelines of the Animal Care and Use Committee of Shandong First Medical University.

As to the mild NASH mouse models, 8-week-old male C57BL/6J mice were fed with a high-fat and high-cholesterol diet (21.1% fat, 14% protein and 2% cholesterol, Nantong Trophic Diet Technology Company, TP 26300) and with a high sugar solution (23.1 g/L D-fructose, (Aladdin, F108332) and 18.9 g/L D-glucose (Aladdin, G116303)) for the whole prevention period (16 weeks). The mice in the normal control group (Chow) were fed with a normal chow diet and with normal distilled water for the whole period (16 weeks).

As to the moderate NASH mouse models, 8-week-old male C57BL/6J mice were fed with a western diet (21.1% fat, 41% sucrose and 1.25% cholesterol, Beijing KeAo Xielie Feed Co., LTD) and with a high sugar solution (23.1 g/L D-fructose, (Aladdin, F108332) and 18.9 g/L D-glucose (Aladdin, G116303)) for 20 weeks, and were injected with CCl_4_ (Aladdin, C131583) at a dose of 0.2 μL/g body weight twice a week during the beginning 12 weeks before treatment 3. The mice in the normal control group (LAD) were fed with a normal chow diet and with normal distilled water for the whole period (20 weeks).

### *In vivo* hydrogen capture and catalytic ·OH hydrogenation

As to the liver-targeted accumulation, eight-week-old male C57BL/6J mice were intravenously injected with 10 mg/kg Pd nanoparticles, and their main organs were extracted at fixed time points for Pd content analysis by ICP technique. In addition, ultrathin liver sections at 1 week post injection were visualized under a Hitachi TEM system (HT7800) after negative staining.

The hydrogen capture *in vivo* was determined by *in situ* measuring H_2_ concentration in the liver of moderate NASH mice using the hydrogen microelectrode (Unisense, Aarhus N, Denmark) 50. The NASH mice were intravenously injected with 10 mg/kg Pd particles, and 12 h later, were subjected to 4% hydrogen gas inhalation for 3 h. Mice were immediately anesthetized by intraperitoneal injection of 7 mL/kg 20% urethane after hydrogen gas inhalation. A midline incision on the abdomen was made to expose liver, and the Clark-type hydrogen microelectrode was inserted 1 mm into the liver to detect local H_2_ concentration in real time. The whole procedure of anesthesia and microeletrode stabilization took only 3 min and was consistent among individuals. Only hydrogen gas inhalation without Pd injection was used a control to distinguish the local hydrogen capture ability of Pd nanoparticles in the liver.

The catalytic ·OH hydrogenation *in vivo* was determined by measuring ·OH and ROS levels in the isolated liver of NASH mice using corresponding kits. The moderate NASH mice were intravenously injected with 10 mg/kg Pd nanoparticles, and after 12 h, were subjected to 4% hydrogen gas inhalation for 3 h. Mice were immediately euthanatized, and fresh liver samples were collected for measurement of ·OH and ROS levels. HPF (10 μM, Invitrogen™, H36004) was added in 200 μL liver lysate and the fluoresce at 490/515 nm was detected under a microplate reader (Molecular Devices i3x) to determine the level of ·OH. The ROS level in liver was tested using a commercial kit (BioLab, Beijing, HR8835). Protein concentrations were determined using a BCA kit (Solarbio, Beijing, PC0020) for normalization.

### Mild NASH prevention experiment

The mild NASH mice were randomly divided into 4 groups (*n* = 6), namely HFHC, HFHC+PdH, HFHC+H_2_, and HFHC+H_2_+PdH. The mice in the HFHC+PdH and HFHC+H_2_ groups accepted a single dose of intravenous injection of 10 mg/mL PdH nanoparticles at the beginning of the experiment and daily 4% hydrogen gas inhalation for 3 h, respectively, while the mice in the HFHC+H_2_+PdH groups were intravenously injected with 10 mg/mL PdH nanoparticles at the beginning of the experiment followed by daily 3-h 4% hydrogen gas inhalation. At 16 weeks post treatment, serum samples were collected, and mice were euthanatized for various analyses.

### Moderate NASH treatment experiment

The moderate NASH mice were divided into 4 groups (*n* = 6), namely NASH, NASH+PdH, NASH+H_2_, and NASH+H_2_+PdH. Treatment began at 12 weeks after the moderate NASH model was established. The mice in the NASH+PdH and NASH+H_2_ groups were injected intravenously with a single dose of 10 mg/kg PdH nanoparticles and inhaled 4% hydrogen gas for 3 h/day, respectively, while the mice in the NASH+H_2_+PdH group were subjected to intravenous injection of 10 mg/mL PdH nanoparticles at the beginning of the experiment followed by daily 3-h 4% hydrogen gas inhalation. After treatment for 8 weeks, serum samples were collected, and mice were euthanatized for various analyses.

### Serum measurements

The levels of serum inflammatory cytokines including IL-1β, IL-6 and TNF-α were measured with corresponding commercial ELISA kits (Mlbio, Shanghai). Serum lipid levels were tested using commercial kits (Biosino, Beijing) for TC, TG, HDL-C and NEFA. Non-HDL-C was calculated by subtracting HDL-C from TC. Serum samples were tested using commercial kits for AST and ALT (Nanjing Jiancheng Bioengineering Institute, C010-2-1, C009-2-1), glucose (Biosino, Beijing) and insulin (Mlbio, Shanghai).

### Glucose tolerance test (GTT) and insulin tolerance test (ITT)

For GTT, the NASH mice were fasted for 6 h and then given a gavage of 2 g/kg glucose in aqueous solution. Blood samples were taken from the tail vein at 0, 15, 30, 60, and 120 min after administration. Blood glucose levels were measured by a glucometer (Aikang Biotechnology Hangzhou, China). For ITT, mice were fasted for 4 h and then intraperitoneally injected with 0.75 IU/kg insulin (Beyotime Biotechnology, Shanghai, China). Blood glucose levels were measured from the tail vein at 0, 15, 30, 60, and 120 min after insulin administration by the glucometer.

### Liver lipid, ROS, SOD, MDA and GSH assessment

Liver samples were lysed and tested for TC and TG using corresponding commercial kits (Nanjing Jiancheng Bioengineering Institute, A111-1-1, A110-1-1). The levels of MDA (Solarbio, BC0025), ROS (BioLab, Beijing, HR8835), SOD (Nanjing Jiancheng Bioengineering Institute, A001-3-2) and GSH (Beyotime, Beijing, S0057S) in lysed liver samples were measured using corresponding commercial kits according to the manufacturers' instruction. Protein concentrations were determined using the BCA kit (Solarbio, Beijing, PC0020) for normalization.

### Histopathological analysis

Dissected liver samples were immediately fixed within 4% paraformaldehyde and embedded in paraffin after dehydration in gradient ethyl alcohol. Sections (7 μm) were cut for hematoxylin-eosin (H&E) and Masson's trichrome stainings according to standard protocols. The histological scores were evaluated blindly by a professional clinical pathologist. For the Oil Red O staining, liver samples were frozen in optimum cutting temperature compound (OCT). Sections (8 μm) were cut and stained with 0.5% Oil Red O for 30 min at room temperature. Sections were visualized and images were captured using a microscope (Olympus, BX53).

Paraffin-embedded liver sections (7 μm) were dehydrated with xylene and gradient ethyl alcohol. After heat-induced antigen retrieval and blockage of endogenous peroxidase with 3% hydrogen peroxide, sections were incubated with 10% goat serum. Primary antibodies against α-SMA (Proteintech, 14395-1-AP, 1:8000), CD68 (Abcam, Ab125212, 1:200) and F4/80 (Abcam, Ab6640, 1:200) were applied followed by treatment with HRP-, Cy3- and FITC-conjugated antibodies. Sections were visualized and images were captured under the microscope (Olympus, BX53).

### Ultrasound analysis of the liver

Mice were anesthetized with 1-2% isoflurane in oxygen (R510P, RWD, Shenzhen, China) and placed on a heating pad. Abdominal fur was shaved, and a 30 MHz linear array transducer MS-400 was placed under the rib cage with slight pressure. Liver was scanned in B-mode using the 3-D motor on a Vevo 3100 system (FUJIFILM Visual Sonics Inc.). System was set on liver mode, 79 fps, 60 dB, at 12 mm depth and 15.36 mm width. The optimal region-of-interest (ROI) plane was selected, and the renal cortex was used as internal reference to normalize signals in B-mode ultrasound. Images were acquired and analyzed using a Vevo LAB 326 software.

### Transcriptome analysis

For gene expression analysis, liver samples from groups of Chow, HFHC, HFHC+H_2_ and HFHC+H_2_+PdH (*n* = 4) in the mild NASH were dissected and immediately frozen in liquid nitrogen after euthanasia. All the RNAs were extracted, and transcriptome library was constructed at BGI-Shenzhen. Clean reads were aligned to a mouse reference genome GCF_000001635.26_GRCm38.p6. The functional enrichment analysis of differentially expressed genes (DEGs), including Kyoto Encyclopedia of Gene and Genomes (KEGG), pathway enrichment analysis were performed using Dr. Tom, an online analysis system of BGI. The images of Venn diagram, KEGG and heatmap plots were performed using the OmicStudio tools at an open website (https://www.omicstudio.cn).

### Statistical analysis

All the data were presented as mean ± standard deviation (Mean ± SD) with individual data. Student *t*-test was applied for comparison between groups. Difference was considered significant using asterisk as follows: *p < 0.05, **p < 0.01, ***p < 0.001, and ****p < 0.0001. Statistical analysis was performed using SPSS 20.0 software. Figures were designed using Origin 9.1 and Graphpad Prism 8.0. Some schematics were partially created with Biorender.com.

## Supplementary Material

Supplementary figures and tables.
