# Supplementary material for: A strategy of local hydrogen capture and catalytic hydrogenation for enhanced therapy of chronic liver diseases
Source: Theranostics. 2023 Apr 23;13(8):2455–70. doi: 10.7150/thno.80494 (PMC10196827; doi:10.7150/thno.80494)
Supplement: Supplementary file 1 — Supplementary figures and tables. [file thnov13p2455s1.pdf]

Supporting Information

A strategy of local hydrogen capture and catalytic hydrogenation for enhanced therapy of chronic liver diseases

Geru Tao[1,2#], Feng Liu[1,2#], Zhaokui Jin[3#], Boyan Liu[1,2], Hao Wang[1,2], Daosheng Li[4], Wei Tang,[5] Yuan Chen[1,2], Qianjun He[6,7]*, Shucun Qin[1,2]*

1. Key Laboratory of Major Diseases and Hydrogen Medical Translational Applications in Universities of Shandong Province & Key Laboratory of Hydrogen Biomedical Research of Health Commission of Shandong Province The Second Affiliated Hospital of Shandong First Medical University & Shandong Academy of Medical Sciences, Jinan, China.

2. Taishan Institute for Hydrogen Biomedical Research, Shandong First Medical University & Shandong Academy of Medical Sciences, Tai'an, China.

3. School of Biomedical Engineering, Guangzhou Medical University, Guangdong, 511495 China.

4. Pathology department of Tai'an City Central Hospital, Tai'an, China.

5. Key Laboratory of Human-Machine-Intelligence Synergic System, Research Center for Neural Engineering, Shenzhen Institutes of Advanced Technology, Chinese Academy of Sciences, Shenzhen 518055, Guangdong, China

6. Shanghai Key Laboratory of Hydrogen Science & Center of Hydrogen Science, School of Materials Science and Engineering, Shanghai Jiao Tong University, Shanghai 200240, China

7. Shenzhen Research Institute, Shanghai Jiao Tong University, Shenzhen 518057, China

*** Correspondence:**

Shucun Qin, 2 Yingsheng East Road, Tai'an City, Shandong Province, China.
Email: 13583815481@163.com

Qianjun He, 800 Dongchuan Road, Minhang District, Shanghai, China.
E-mail: nanoflower@126.com

[#] These authors contributed equally to this work.

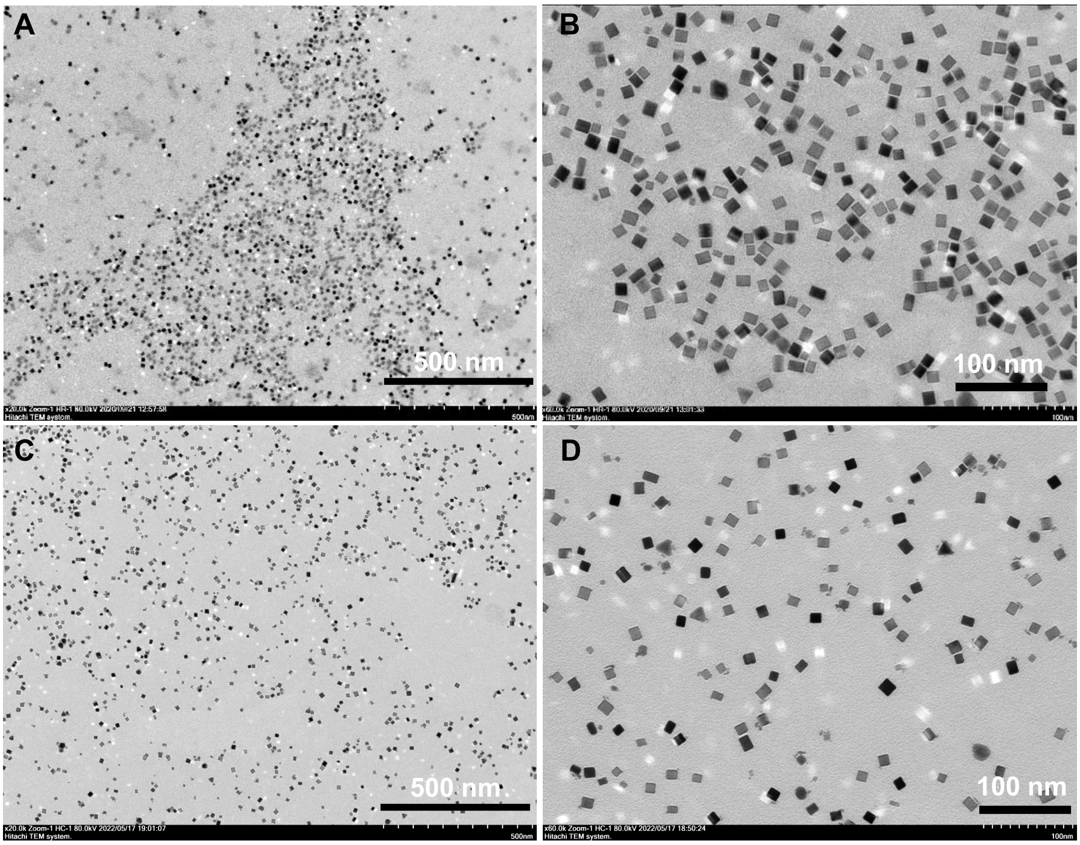

Figure S1. TEM images of Pd (**A**,**B**) and PdH (**C**,**D**) nanoparticles at different magnification folds.

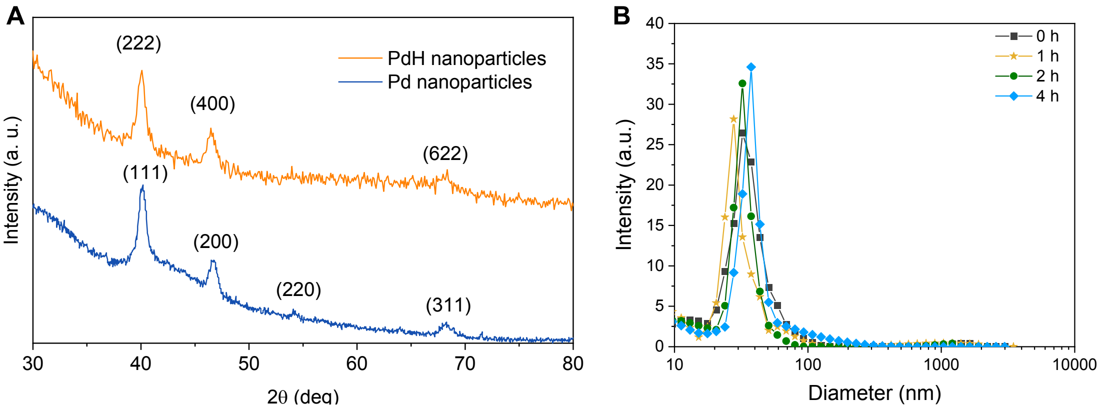

Figure S2. (**A**) XRD patterns of Pd and PdH nanoparticles. The width of characteristic peaks in PdH nanoparticles is slightly bigger than that in Pd nanoparticles, indicating the hydrogen incorporation-induced worse crystallinity of PdH nanoparticles. (**B**) DLS data of PdH nanoparticles dispersed in serum for different time durations.

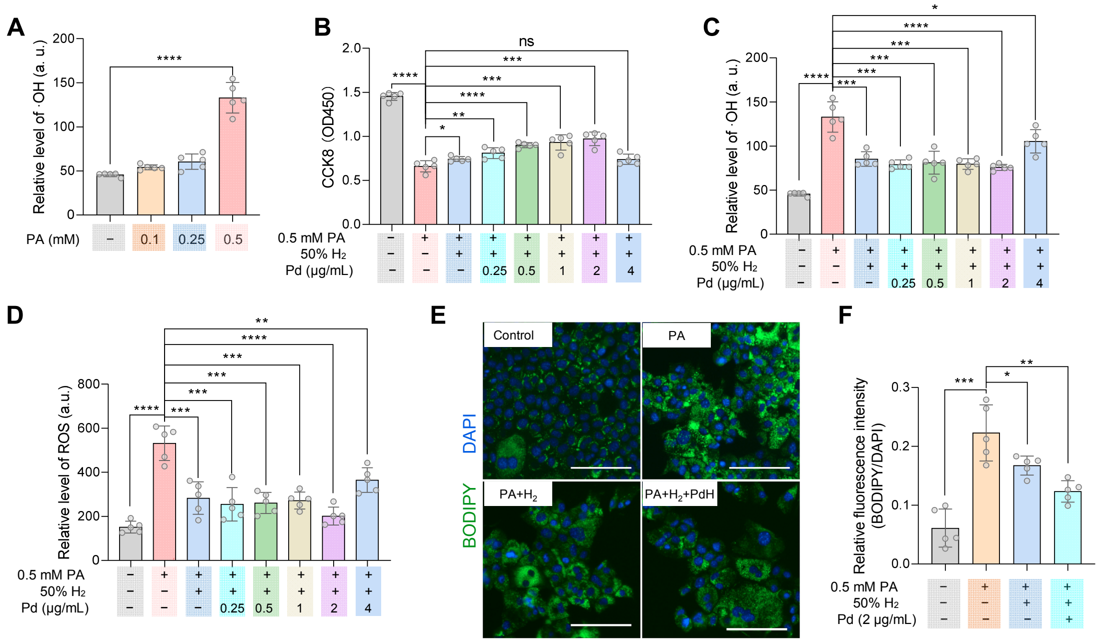

Figure S3. Intracellular hydroxyl radical level in AML-12 cells treated with different concertations of PA (**A**), AML-12 cell proliferation treated with 0.5 mM PA and different concentrations of Pd and/or hydrogen molecules (**B**), **i**ntracellular hydroxyl radical (**C**) and ROS (**D**) levels in PA-treated AML-12 cells incubated with different concentrations of Pd, intracellular lipid level detected by BODIPY staining in 2 μg/mL Pd and 0.5 mM PA-treated AML-12 cells (**E**), and corresponding quantification analysis (**F**). Bar, 100 μm. AML-12 cells were incubated with PA and Pd nanoparticles in the 50% hydrogen incubator for 12 h, and then in the normal incubator without hydrogen gas for another 10 h. All the data were presented as mean ± standard deviation (Mean ± SD) with individual data. Student *t*-test was applied for comparison between groups. Difference was considered significant using asterisk as follows: *$p < 0.05$, **$p < 0.01$, ***$p < 0.001$, and ****$p < 0.0001$.

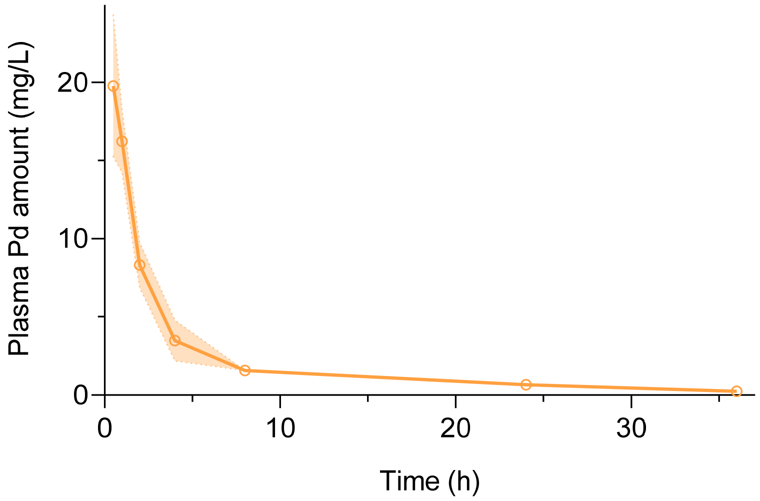

Figure S4. Pharmacokinetic curve of Pd nanoparticles in the blood (n=3).

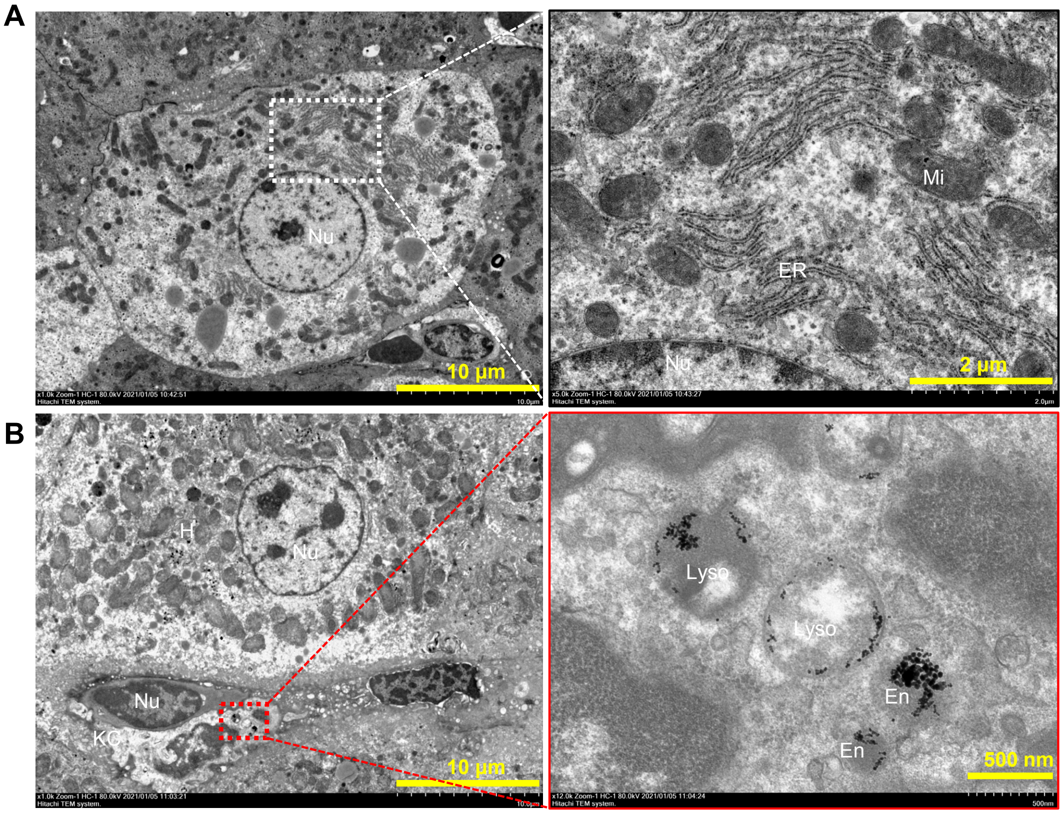

Figure S5. TEM images of liver sections extracted from the mice at 1 week post intravenous injection with Pd nanoparticles. Hepatocyte (**A**), Kupffer cell (**B**). H, hepatocyte; KC, Kupffer cell; Nu, nuclear; ER, endothelium reticulum; Mi, mitochondria; Lyso, lysosome; En, endosome.

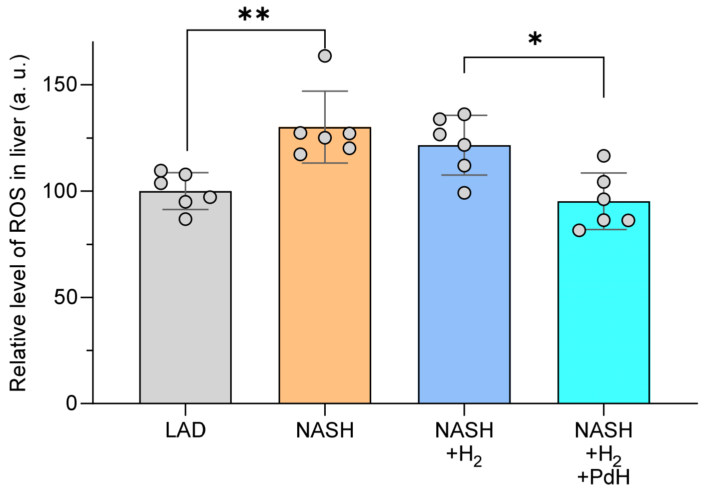

Figure S6. The ROS level in the normal (LAD) and NASH livers after various treatments. Data were presented as mean ± standard deviation (Mean ± SD) with individual data. Student *t*-test was applied for comparison between groups. Difference was considered significant using asterisk as *$p < 0.05$, **$p < 0.01$.

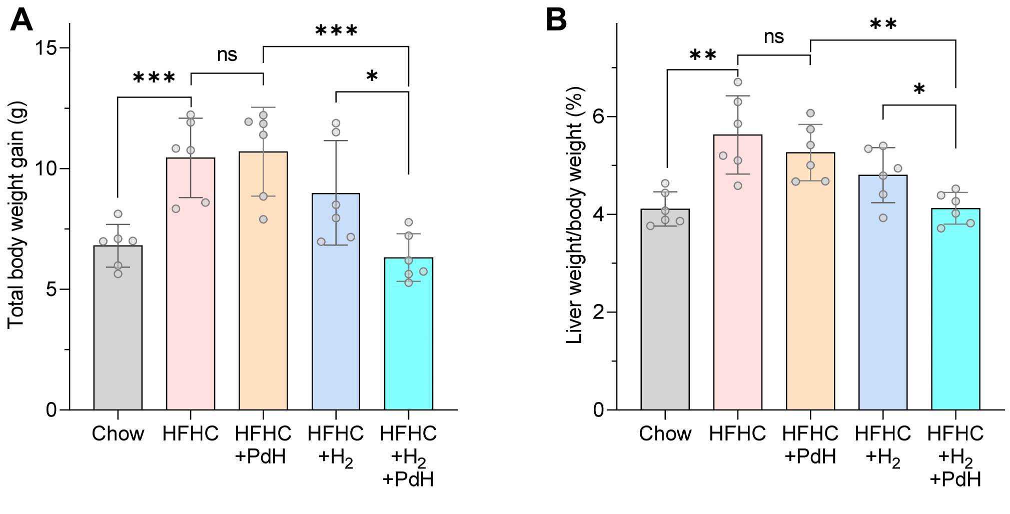

Figure S7. Total body weight gain (**A**) and the ratio of liver weight to body weight (**B**) in the prevention of NASH. All the data were presented as mean ± standard deviation (Mean ± SD) with individual data. Student *t*-test was applied for comparison between groups. Difference was considered significant using asterisk as follows: *$p < 0.05$, **$p < 0.01$, ***$p < 0.001$.

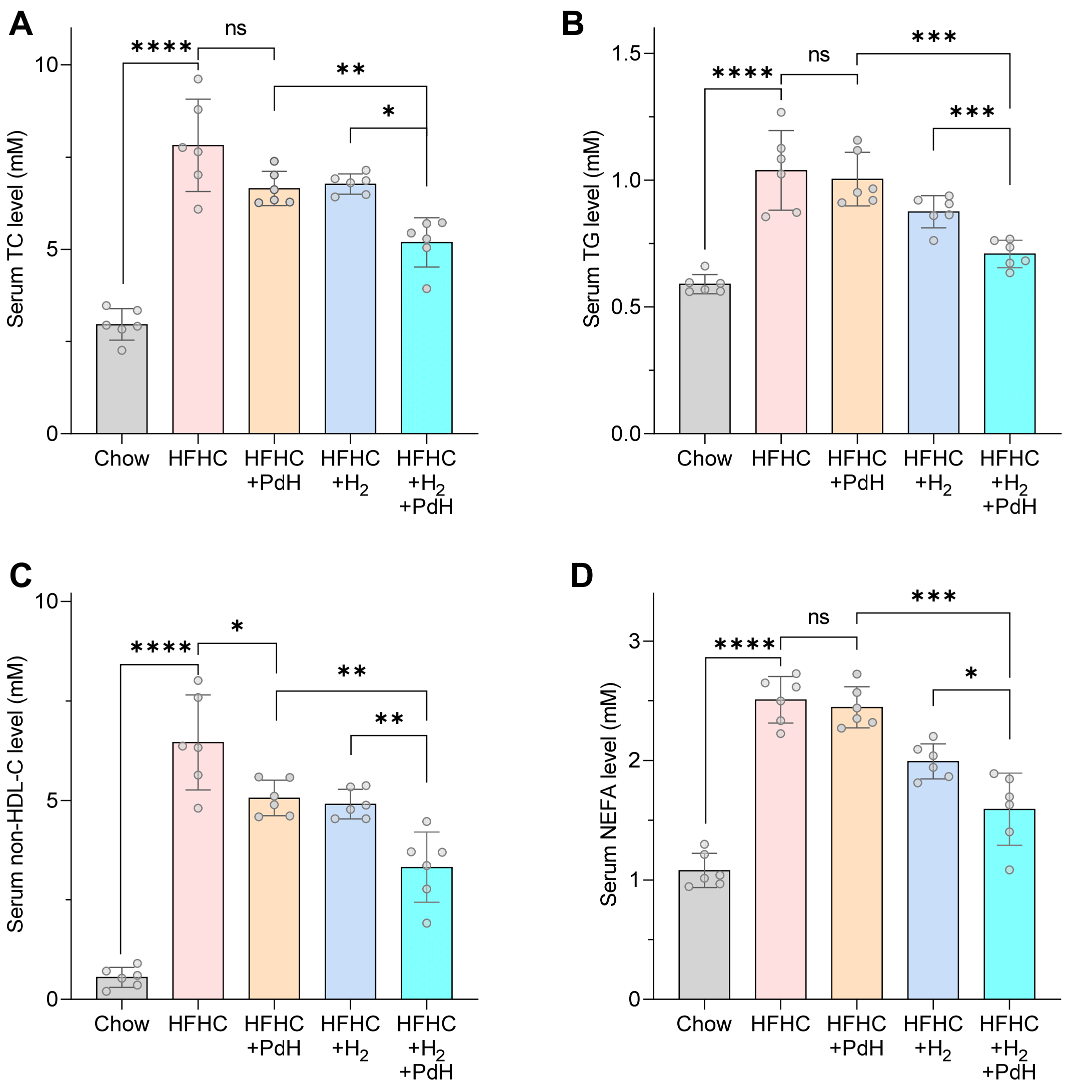

Figure S8. Levels of serum lipids including TC (**A**), TG (**B**), non-HDL-C (**C**) and NEFA (**D**) in the prevention of NASH. Data were presented as mean ± standard deviation (Mean ± SD) with individual data. Student *t*-test was applied for comparison between groups. Difference was considered significant using asterisk as follows: *$p < 0.05$, **$p < 0.01$, ***$p < 0.001$ and ****$p < 0.0001$.

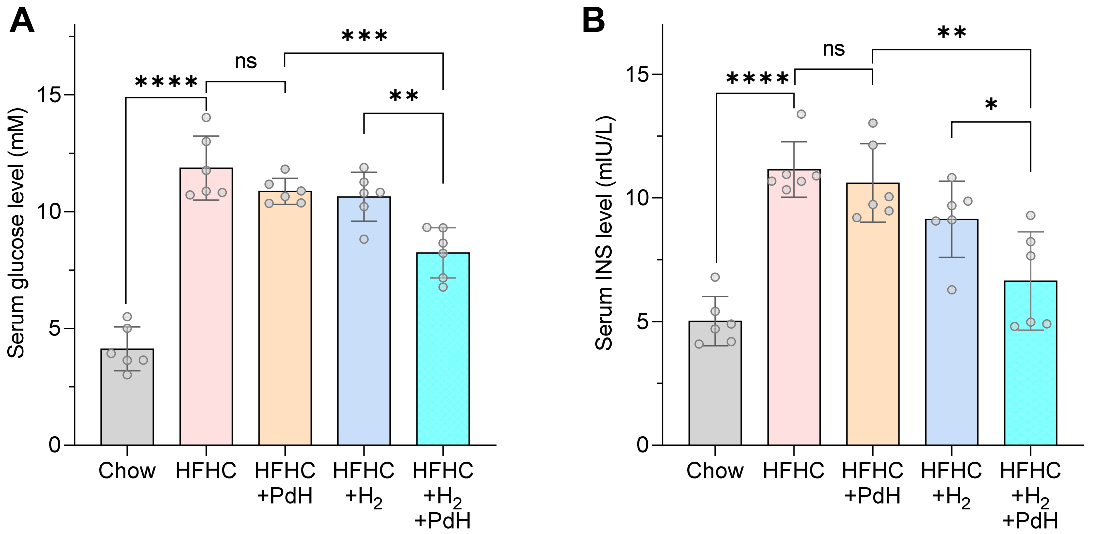

Figure S9. Levels of serum glucose (**A**) and insulin (**B**) in the prevention of NASH. Data were presented as mean ± standard deviation (Mean ± SD) with individual data. Student *t*-test was applied for comparison between groups. Difference was considered significant using asterisk as follows: *$p < 0.05$, **$p < 0.01$, ***$p < 0.001$ and ****$p < 0.0001$.

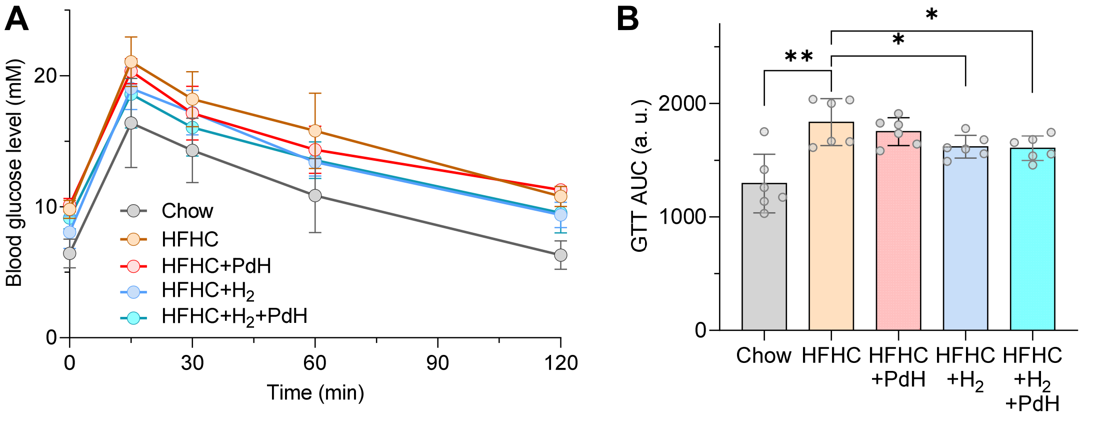

Figure S10. Glucose tolerance test (GTT): blood glucose level (**A**) and corresponding quantitative results of areas under curve (**B**) in the prevention of NASH. Data were presented as mean ± standard deviation (Mean ± SD) with individual data. Student *t*-test was applied for comparison between groups. Difference was considered significant using asterisk as follows: *$p < 0.05$, **$p < 0.01$.

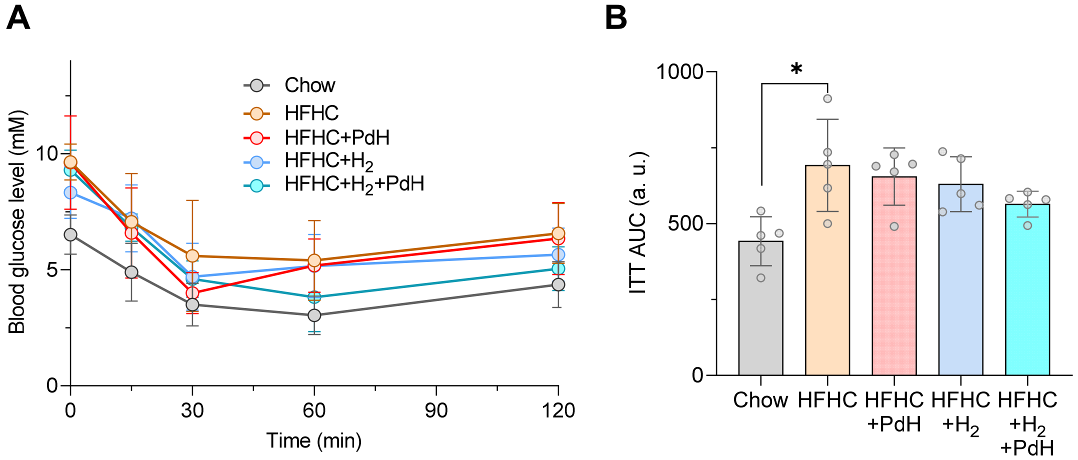

Figure S11. Insulin resistant test (ITT): blood glucose level (**A**) and corresponding quantitative results of areas under curve (**B**) in the prevention of NASH. Data were presented as mean ± standard deviation (Mean ± SD) with individual data. Student *t*-test was applied for comparison between groups. Difference was considered significant using asterisk as follows: *$p < 0.05$.

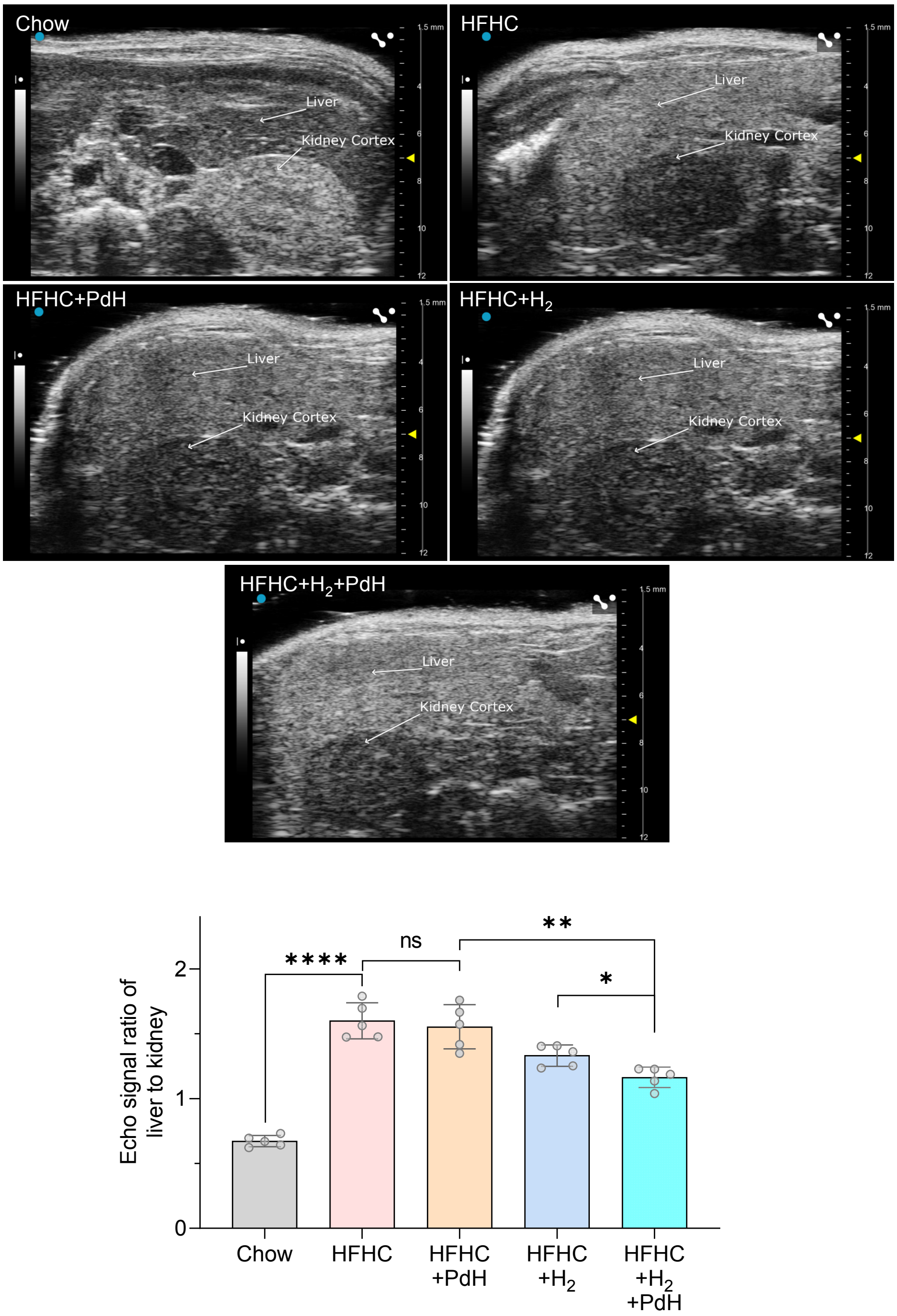

Figure S12. B-mode ultrasound analysis of mice liver lipid contents in the prevention of NASH. The ultrasound echo signals of ROI in the liver and renal cortex were determined on the Vevo3100 system, and the hepatic/renal signal ratio was used as an indicator of liver lipid content. Data were presented as mean ± standard deviation (Mean ± SD) with individual data. Student *t*-test was applied for comparison between groups. Difference was considered significant using asterisk as follows: *$p < 0.05$, **$p < 0.01$, **$p < 0.01$, ***$p < 0.001$ and ****$p < 0.0001$.

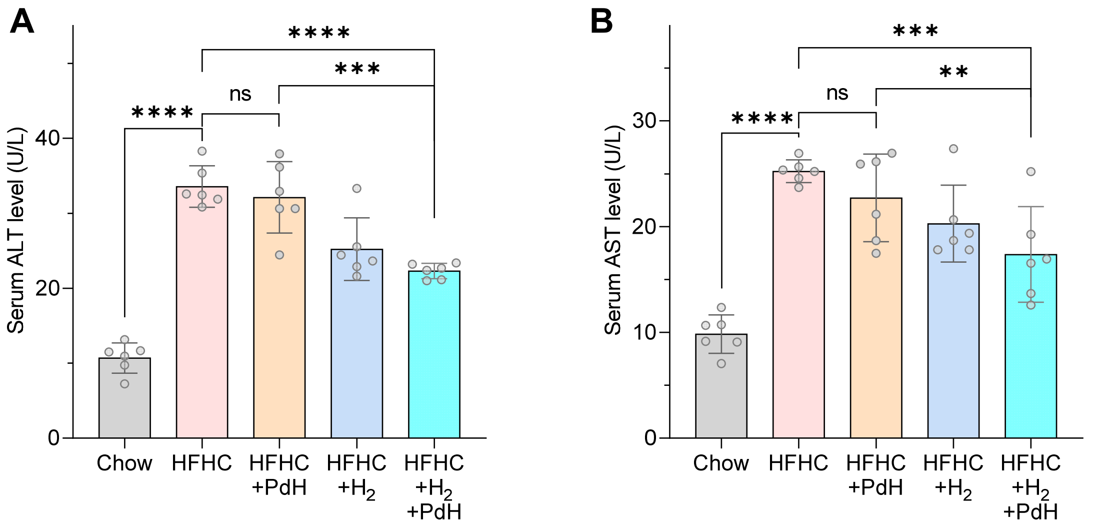

Figure S13. Levels of liver enzymes including ALT (**A**) and AST (**B**). Data were presented as mean ± standard deviation (Mean ± SD) with individual data. Student *t*-test was applied for comparison between groups. Difference was considered significant using asterisk as follows: *$p < 0.05$, **$p < 0.01$, ***$p < 0.001$, and ****$p < 0.0001$.

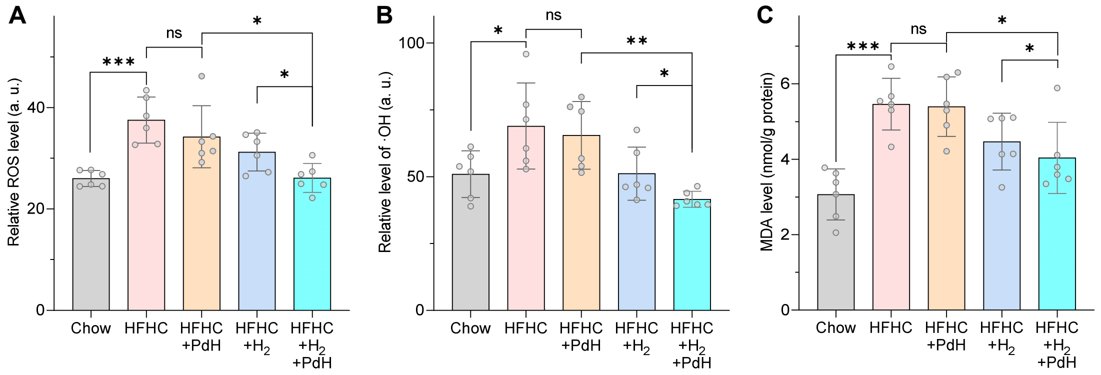

Figure S14. Oxidative stress levels in liver in the prevention of NASH: ROS (**A**),·OH (**B**), and MDA (**C**).Data were presented as mean ± standard deviation (Mean ± SD) with individual data. Student *t*-test was applied for comparison between groups. Difference was considered significant using asterisk as follows: *$p < 0.05$, **$p < 0.01$, ***$p < 0.001$.

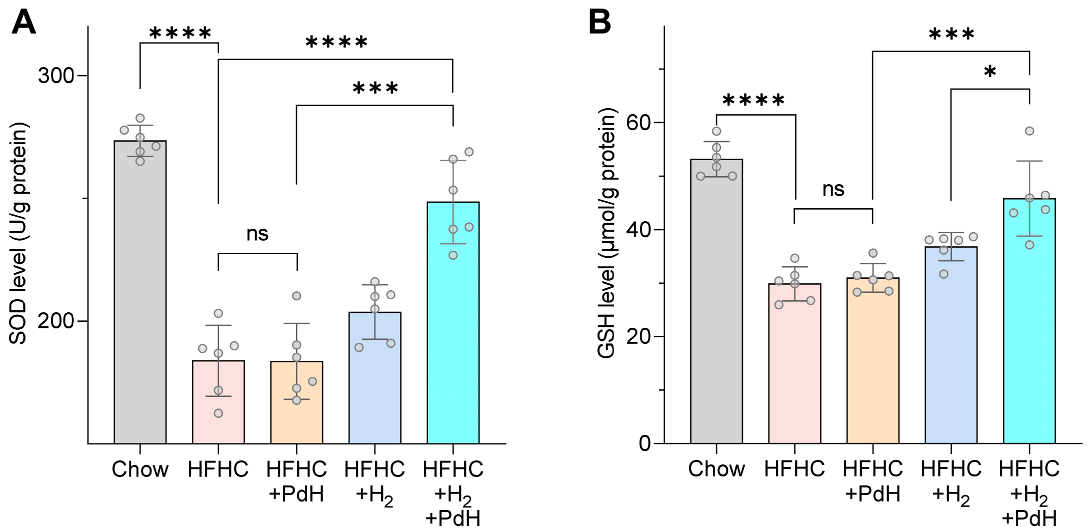

Figure S15. Anti-oxidative capability of mice liver in the prevention of NASH: SOD level (**A**), and GSH level (**B**). Data were presented as mean ± standard deviation (Mean ± SD) with individual data. Student *t*-test was applied for comparison between groups. Difference was considered significant using asterisk as follows: *$p < 0.05$, **$p < 0.01$, ***$p < 0.001$, and ****$p < 0.0001$.

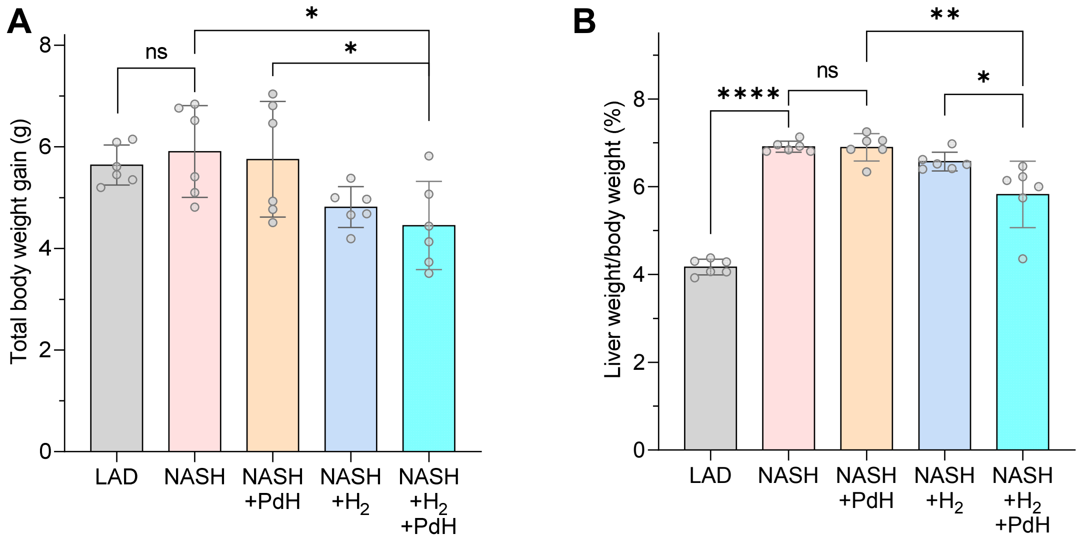

Figure S16. Total weight gain (**A**) and the liver/body ratio (**B**) in the NASH treatment. Data were presented as mean ± standard deviation (Mean ± SD) with individual data. Student *t*-test was applied for comparison between groups. Difference was considered significant using asterisk as follows: *$p < 0.05$, **$p < 0.01$, ***$p < 0.001$ and ****$p < 0.0001$.

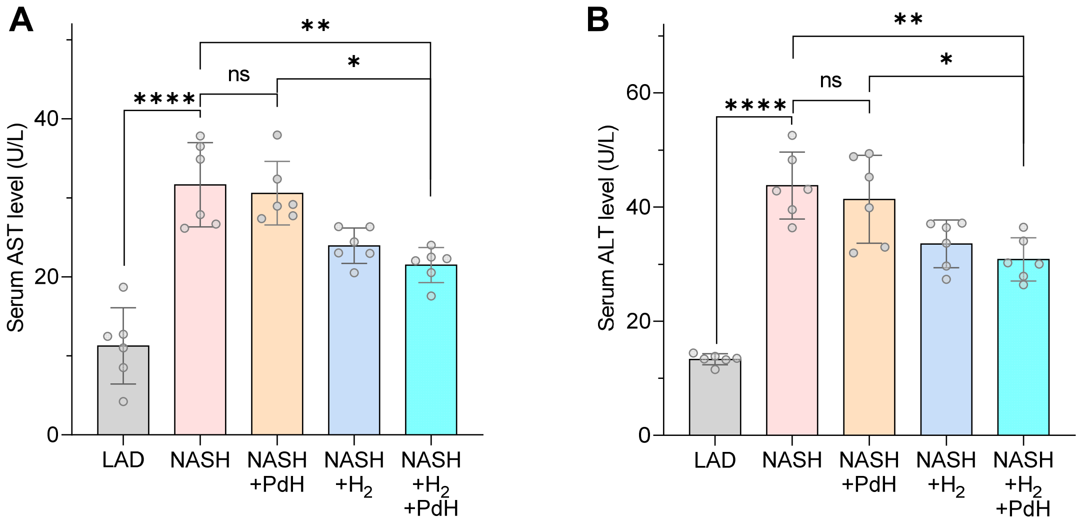

Figure S17. Levels of liver enzymes ALT (**A**) and AST (**B**) in the treatment of NASH. Data were presented as mean ± standard deviation (Mean ± SD) with individual data. Student *t*-test was applied for comparison between groups. Difference was considered significant using asterisk as follows: $*p < 0.05$, $**p < 0.01$, $***p < 0.001$ and $****p < 0.0001$.

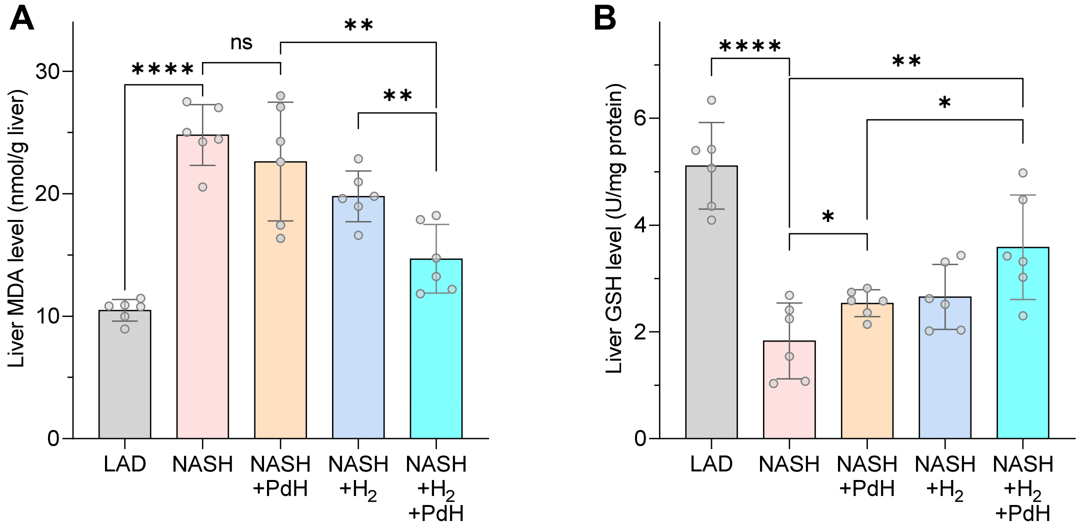

Figure S18. Oxidative stress level using MDA (**A**) and anti-oxidative capacity using GSH (**B**) as indicators of mice liver in the treatment of NASH. Data were presented as mean ± standard deviation (Mean ± SD) with individual data. Student *t*-test was applied for comparison between groups. Difference was considered significant using asterisk as follows: $*p < 0.05$, $**p < 0.01$, $***p < 0.001$ and $****p < 0.0001$.

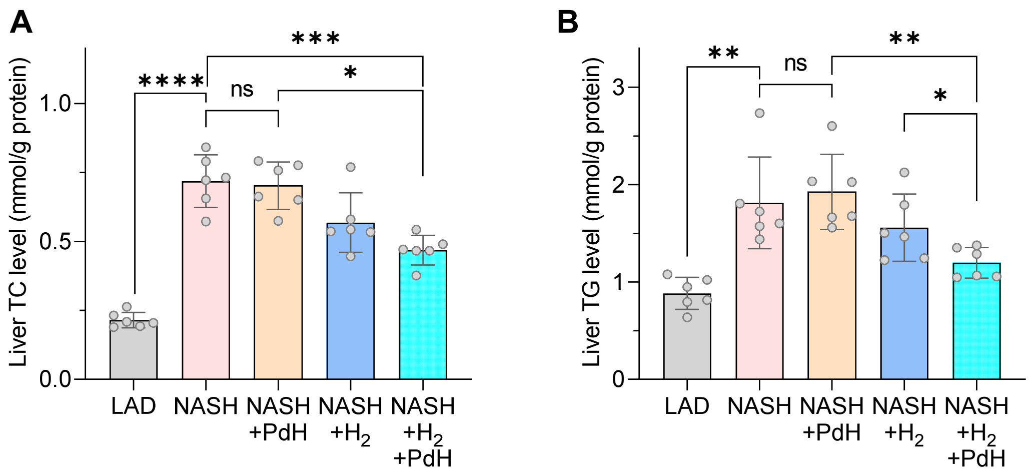

Figure S19. The TC (**A**) and TG (**B**) levels in liver in the treatment of NASH. Data were presented as mean ± standard deviation (Mean ± SD) with individual data. Student *t*-test was applied for comparison between groups. Difference was considered significant using asterisk as follows: $*p < 0.05$, $**p < 0.01$, $***p < 0.001$, $***p < 0.001$ and $****p < 0.0001$.

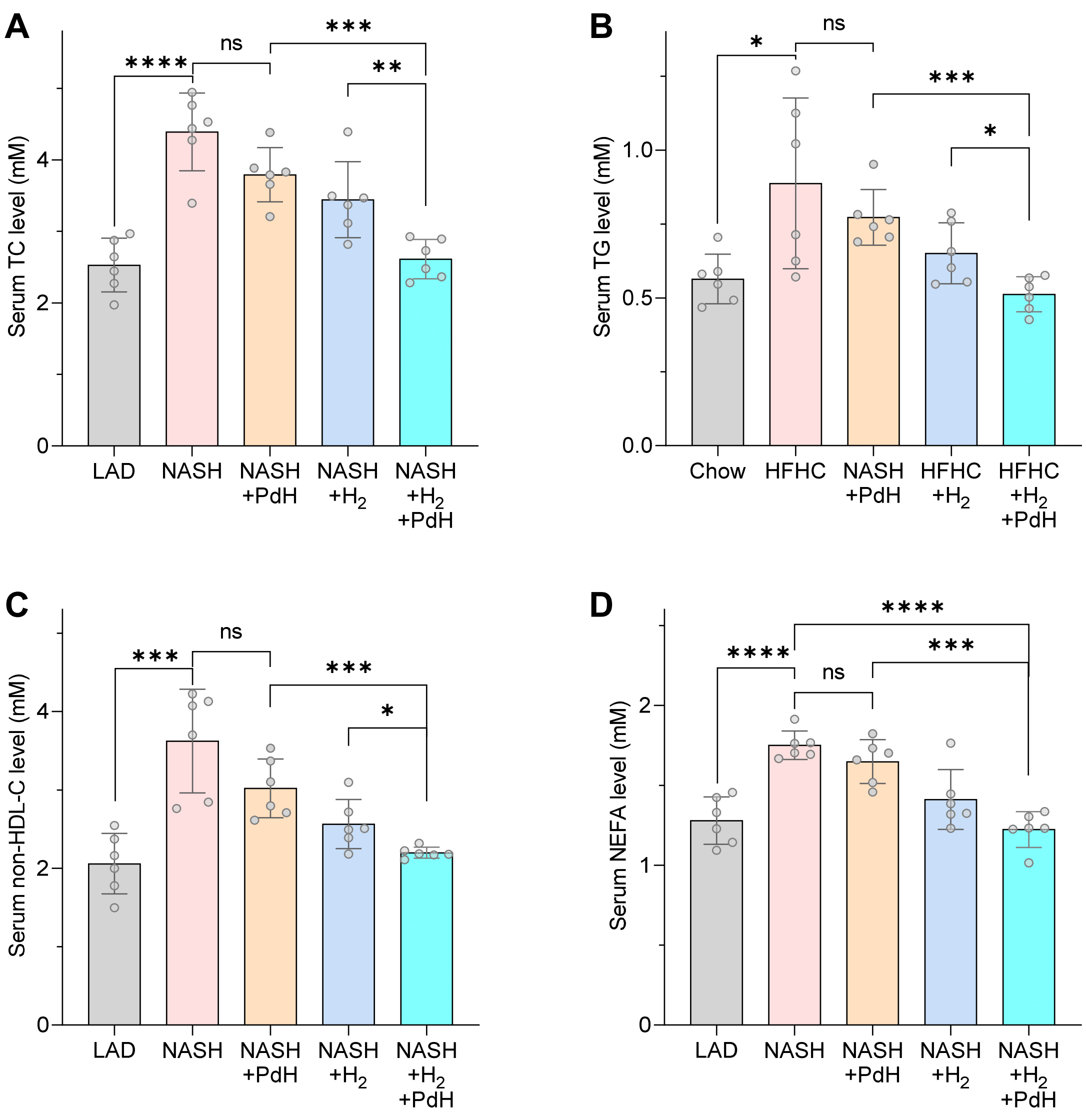

Figure S20. Levels of serum lipids including TC (**A**), TG (**B**), non-HDL-C (**C**), and NEFA (**D**) in the treatment of NASH. Data were presented as mean ± standard deviation (Mean ± SD) with individual data. Student *t*-test was applied for comparison between groups. Difference was considered significant using asterisk as follows: *$p < 0.05$, **$p < 0.01$, ***$p < 0.001$ and ****$p < 0.0001$.

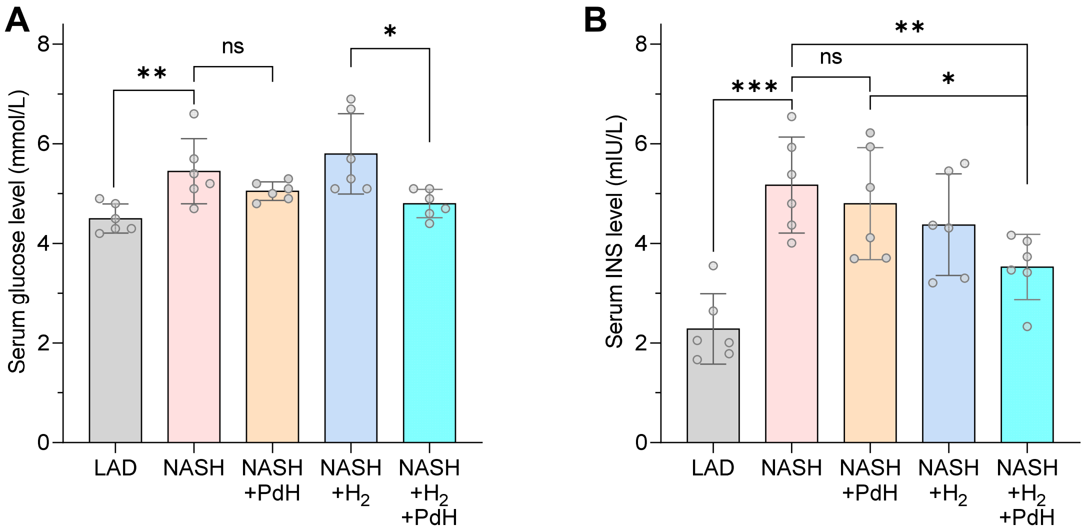

Figure S21. Serum glucose (**A**) and insulin (**B**) levels in the treatment of NASH. Data were presented as mean ± standard deviation (Mean ± SD) with individual data. Student *t*-test was applied for comparison between groups. Difference was considered significant using asterisk as follows: *$p < 0.05$, **$p < 0.01$, ***$p < 0.001$.

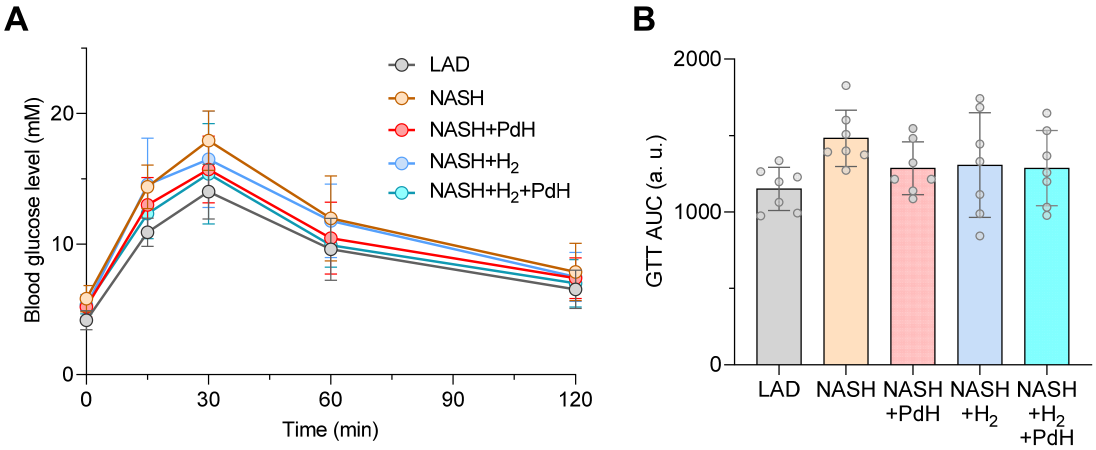

Figure S22. Glucose tolerance behaviors: blood glucose level (**A**) and corresponding quantitative results of areas under curve (**B**) in the treatment of NASH. Data were presented as mean ± standard deviation (Mean ± SD) with individual data. Student *t*-test was applied for comparison between groups.

Table S1. CLD activity score of liver histology in the moderate NASH treatment.

Group	Steatosis			Lobular inflammation			Ballooning		
	S2 No.	S3 No.	Mean	L1 No.	L2 No.	Mean	B1 No.	B2 No.	Mean
LAD	0/6	0/6	0.00	3/6	0/6	0.50	5/6	0/6	0.83
NASH	4/6	2/6	2.33	1/6	5/6	1.83	5/6	1/6	1.17
NASH +PdH	4/6	2/6	2.33	2/6	4/6	1.67	5/6	1/6	1.17
NASH +H_2	4/6	2/6	2.33	3/6	3/6	1.50	5/6	1/6	1.17
NASH +H_2+PdH	5/6	1/6	2.17	5/6	1/6	1.1	6/6	0/6	1.00

Note: S2 No. and S3 No. represent the number of steatosis with 2 and 3 scores, respectively. L1 No. and L2 No. represent the number of lobular inflammation with 1 and 2 scores, respectively. B1 No. and B2 No. represent the number of ballooning with 1 and 2 scores, respectively.

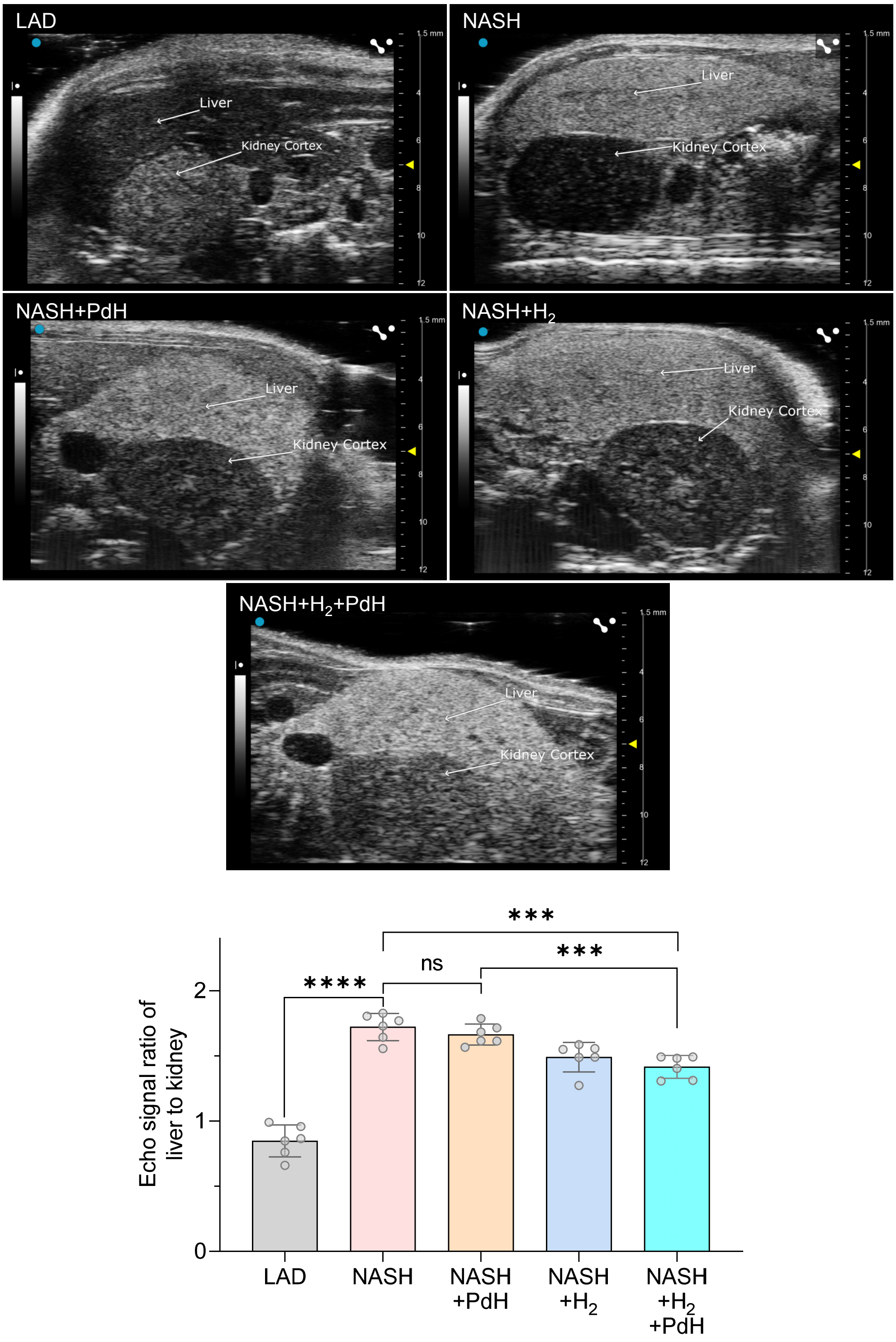

Figure S23. B-mode ultrasound analysis of mice liver lipid contents in the treatment of NASH. The hepatic/renal signal ratio was used as an indicator of liver lipid content. Data were presented as mean ± standard deviation (Mean ± SD) with individual data. Student *t*-test was applied for comparison between groups. Difference was considered significant using asterisk as follows: *$p < 0.05$, **$p < 0.01$, ***$p < 0.001$ and ****$p < 0.0001$.

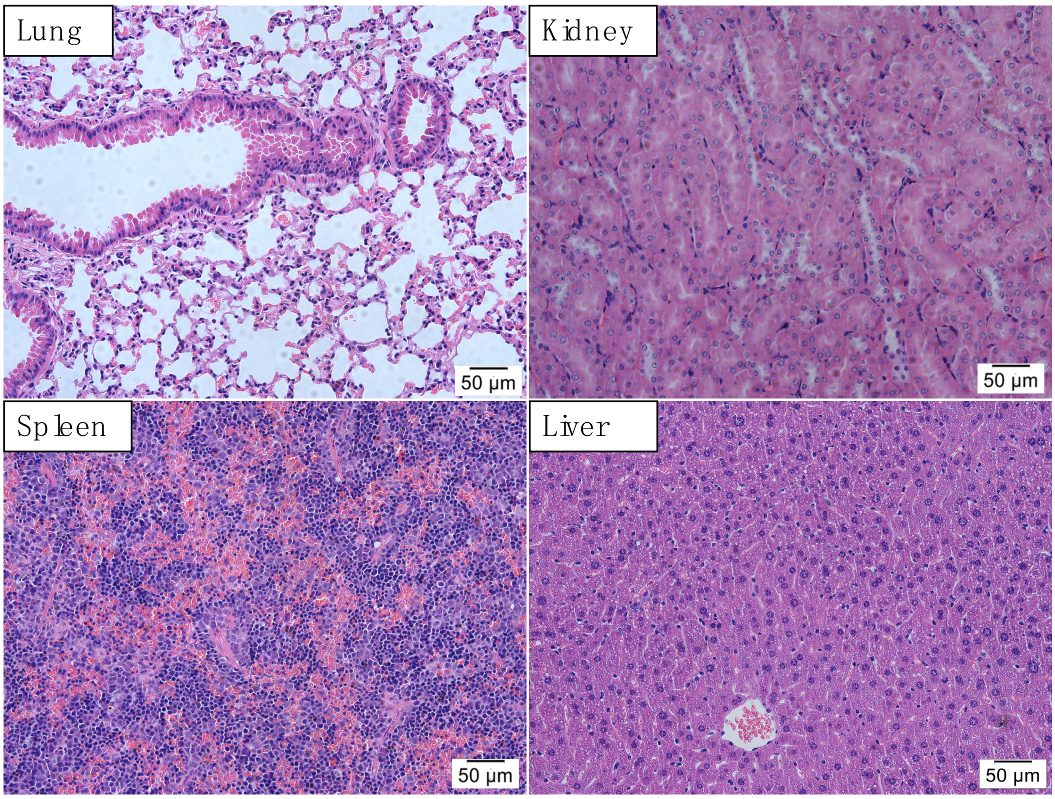

Figure S24. H&E staining of lung, kidney, spleen and liver of mouse injected with 10 mg/kg PdH nanoparticles for 24 weeks.

Table S2 Primers of qPCR analysis.

Name	Primer sequence	Product (bp)
m-Tnf	F: 5'- CCCTCACACTCAGATCATCTTCT- 3'<br>R: 5'- GCTACGACGTGGGCTACAG- 3'	61
m-Il1b	F: 5'- GCAACTGTTCCTGAACTCAACT- 3'<br>R: 5'- ATCTTTTGGGGTCCGTCAACT- 3'	89
m-Il6	F: 5'- TAGTCCTTCCTACCCCAATTTCC- 3'<br>R: 5'- TTGGTCCTTAGCCACTCCTTC- 3'	76
m-Gapdh	F: 5'- AGGTCGGTGTGAACGGATTTG- 3'<br>R: 5'- TGTAGACCATGTAGTTGAGGTCA- 3'	123
h-Tnf	F: 5'- GAGGCCAAGCCCTGGTATG- 3'<br>R: 5'- CGGGCCGATTGATCTCAGC- 3'	91
h-Il1b	F: 5'- ATGATGGCTTATTACAGTGGCAA- 3'<br>R: 5'- GTCGGAGATTCGTAGCTGGA- 3'	132
h-Il6	F: 5'- ACTCACCTCTTCAGAACGAATTG- 3'<br>R: 5'- CCATCTTTGGAAGGTTCAGGTTG- 3'	149
h-Gapdh	F: 5'- GGAGCGAGATCCCTCCAAAAT- 3'<br>R: 5'- GGCTGTTGTCATACTTCTCATGG- 3'	197
